# NEAT1 promotes the malignant development of bladder cancer by regulating the miR-101/VEGF-C pathway in vitro and in vivo

**DOI:** 10.1186/s12894-022-01151-z

**Published:** 2022-11-25

**Authors:** Huihui Zhang, Shuang Yu, Kuilin Fei, Zhongxin Huang, Shidong Deng, Hanfeng Xu

**Affiliations:** 1 Department of Urology, The First Affiliated Hospital, Hengyang Medical School, University of South China, 69 Chuanshan Road, Hengyang, 421001 Hunan People’s Republic of China; 2 Institute of Hospital Administration, University of South China, Hengyang, 421001 Hunan People’s Republic of China; 3 Department of Urology, The People’s Hospital of Liuyang, Changsha, 410399 Hunan People’s Republic of China; 4 Department of Obstetrics, Xiangya Hospital, Central South University, Changsha, 410008 Hunan People’s Republic of China

**Keywords:** NEAT1, miR-101, VEGF-C, Bladder cancer

## Abstract

**Background:**

NEAT1 has been shown to play an oncogenic role in many kinds of cancers. However, detailed roles of NEAT1 in bladder cancer are largely unknown.

**Methods:**

In the present study, the expression of NEAT1, miR-101 and VEGF-C was detected in human bladder cancer samples. The relationship between NEAT1 and the prognosis of patients with bladder cancer was analysed. In vitro experiments explored the effects of NEAT1 on biological behaviours of bladder cancer T24 and 5637 cells. Bioinformatics prediction and luciferase assays were used to assay the regulatory mechanism of action of NEAT1 and miR-101. Loss and gain of the expression of miR-101 and VEGF-C were used to explore the effects of the NEAT1/miR-101/VEGF-C pathway on T24 and 5637 cells. The effect of NEAT1 on the growth of bladder cancer in vivo was explored using an orthotopic tumourigenesis model.

**Results:**

NEAT1 and VEGF-C were significantly upregulated in bladder cancer samples, and miR-101 was significantly downregulated. NEAT1 upregulation was associated with poorer recurrence-free survival of patients with bladder cancer. Overexpression of NEAT1 promoted the proliferation, migration and invasion of bladder cancer cells. The results of the luciferase assay indicated that miR-101 was a target of NEAT1. The promoting effects of NEAT1 on bladder cancer cells were reversed by miR-101 upregulation, and inhibition of miR-101 enhanced the effects of NEAT1. Overexpression of VEGF-C had a clear synergistic effect with the action of NEAT1. Overexpression of NEAT1 increased tumour growth and induced the development of liver metastasis.

**Conclusions:**

In conclusion, our data indicated that NEAT1 was expressed at high levels in bladder cancer patients and correlated with unfavourable prognosis. NEAT1 promoted malignant development of bladder cancer in vitro and in vivo by regulating the miR-101/VEGF-C pathway.

**Supplementary Information:**

The online version contains supplementary material available at 10.1186/s12894-022-01151-z.

## Introduction

Bladder cancer (BC) is one of the most common malignant tumours in the urinary system, and BC has a high mortality rate worldwide [1]. The presence of the lymph node metastasis is one of the most significant prognostic factors for patients with bladder cancer. Therefore, identification of the markers of bladder cancer related to metastasis is critical for the investigations of the mechanism.

MicroRNAs (miRNAs) are a class of endogenous small noncoding RNAs implicated in numerous cancers by acting as oncogenes or tumour suppressors [2]. Nuclear enriched abundant transcript 1 (NEAT1) is a lncRNA specifically located in paraspeckles that functions as an essential structural determinant by interacting with the members of the Drosophila behaviour human splicing (DBHS) family of proteins [3]. Recent studies have shown that NEAT1 is upregulated and acts as a tumour promoter gene in various cancers. Li [4] demonstrated that NEAT1 promotes glioma cancer progression via the regulation of miR-98-5p/BZW1. Zhao [5] showed that the downregulation of NEAT1 suppresses the growth, migration and invasion of non-small-cell lung cancer and facilitates apoptosis. Moreover, some studies have indicated that NEAT1 is involved in cisplatin and doxorubicin resistance to BC treatment [6, 7]. However, a recent expression analysis of a panel of long noncoding RNAs (lncRNAs) notably revealed that NEAT1 is significantly downregulated in bladder tumour tissues compared with normal tissues [8]. This result was not consistent with the previously proposed roles for NEAT1. Thus, the significance and mechanism of action of NEAT1 in tumourigenesis of BC are poorly understood. In the present study, a novel mechanism of action of NEAT1 was demonstrated to promote tumour development of BC by modulating the miR-101/VEGF-C pathway, providing novel insight into therapeutic application of lncRNAs in BC.

## Materials and methods

### Clinical study

We retrospectively reviewed the records of patients diagnosed with BC undergoing surgical resection at the First Affiliated Hospital of University of South China between January 2017 and January 2020. A total of 60 patients were enrolled as study subjects. None of these patients underwent preoperative chemoradiotherapy. Tumour tissues were collected, and adjacent normal tissue samples were used as controls. The clinical data and recurrence-free survival of these patients were retrospectively reviewed. The censor date for the recurrence-free survival data was set as a date 48 months after the surgery. The present study was approved by the Ethics Committee of the First Affiliated Hospital of University of South China (No. 20160308), and all patients signed the informed consent forms.

### Cell culture

The human bladder cancer T24 and 5637 cell lines were purchased from the Cell Bank affiliated with Shanghai Institute of Biochemistry and Cell Biology. The cells were cultured in RPMI-1640 medium (Thermo Fisher Scientific, Waltham, MA, USA) containing 10% foetal bovine serum, 100 U/ml penicillin and 100 µg/ml streptomycin. All cells were cultured in 5% CO_2_ at 37 °C.

### Transfection

PCR was used to amplify the full-length cDNA of human NEAT1. The primer sequences used in the present study were as follows: NEAT1: F-GGGGTACCCTTCCTCCCTTTAACTTATCCATTCAC and R-GGAAGCTTCATAACAACCATTACCACCTCCTTCTC. The ectopic vector pcDNA3.1-NEAT1 and the corresponding control vector were constructed. Lipofectamine 3000 (Invitrogen, Grand Island, NY, USA) was used for transient transfection experiments according to the manufacturer’s instructions. The ectopic and control vectors were added to the cells of the NEAT1 and negative control (NC) groups, respectively. T24 and 5637 cells without transfection were used as the control (CON) group. The miR-101 mimic and inhibitor with the corresponding negative controls (miR-101 mimic, miR-101 inhibitor, miR-101 mimic NC and miR-101 inhibitor NC), the VEGF-C overexpression plasmid were purchased from RiboBio (Ribobio, Guangzhou, China).

### QRT-PCR

Total RNA was isolated using TRIzol reagent (Invitrogen, Grand Island, NY, USA). Six microlitres of total RNA was subjected to reverse transcription by using a PrimeScript™ RT reagent kit with gDNA Eraser (Takara, Shiga, Japan). QRT–PCR was performed using the StepOnePlus™ real-rime PCR system (Applied Biosystems, Foster City, CA, USA). The sequences of the PCR primers were as follows: NEAT1: F-CAGTTAGTTTATCAGTTCTCCCATCCA, R-GTTGTTGTCGTCACCTTTCAACTCT; miR-101: F-UACAGUACUGUGAUAACUGAA, R-CAGUUAUCACAGUACUGUAUU; U6: F-CTCGCTTCGGCAGCACA, R-AACGCTTCACGAATTTGCGT; VEGF-C: F-AGAAGGAGGAGGGCAGAAT, R-GTCTCGATTGGATGGCAGTAG; and GAPDH: F-GTGGTCTCCTCTGACTTCAAC, R-CCTGTTGCTGTAGCCAAATTC. The data were recalculated by the 2^–ΔΔCt^ method after normalization against the corresponding endogenous controls (GAPDH and U6).

### Western blot analysis

The cells were lysed in RIPA buffer and centrifuged at 12,000 rpm for 5 min. Then, the supernatant was collected. Protein concentrations were determined using a BCA protein assay (Beyotime Biotechnology, Shanghai, China). Thirty micrograms of protein was processed by electrophoresis and transferred onto polyvinylidene difluoride membranes (Millipore, Bedford, MA, USA). Then, the membranes were blocked using skimmed milk for 1 h. After washing with TBST 3 times for 10 min each time, the membranes were incubated with primary antibodies and then with horseradish peroxidase-conjugated secondary antibodies. The proteins were visualized by enhanced chemiluminescence detection reagent (Thermo Scientific, Waltham, MA, USA). All data were normalized to GAPDH levels. Primary antibodies against VEGF-C (1:1000; ab9546, Abcam, USA) and GAPDH (1:1000; ab8245, Abcam, USA) were used. In the running of experiments, sectioning gels is performed in our research group, as the blots are cut prior to hybridisation with antibodies. So images showing full-length membranes were not provided, but all the replicate versions of original gel image were uploaded on supplemental files to confirm specific detection of the target antigen.

### MTT assay

The cells were cultured in 96-well plates at a density of 5 × 10^4^ cells per well. After overnight incubation, MTT reagent was added to each well, and the plate was incubated for 3 h at 37 °C. Then, the supernatant was removed, and 100 μL of DMSO was added to each well. Absorbance was recorded at a wavelength of 570 nm.

### Migration assay

The cells at a density of 5 × 10^4^ were transferred into the upper Transwell chamber containing serum-free medium. The lower Transwell chamber was filled with medium containing 10% serum. The cells were cultured for 8 h at room temperature. Migrated cells were fixed using 4% paraformaldehyde and stained with 0.1% crystal violet for 10 min. Cell migration was detected, and the number of migrating cells was calculated using an inverted microscope (Nikon, Japan,magnification × 200).

### Invasion assay

For the cell invasion assay, 5 × 10^4^ transfected cells in 200 µL serum-free medium were added to the upper chamber of the Matrigel-precoated inserts (BD Bioscience, USA). The lower chamber was filled with medium supplemented with 10% FBS. After culture in 5% CO_2_ at 37 °C for 8 h, the cells invading into the lower chamber were stained with 0.1% crystal violet (Sigma, USA). PBS was used to wash the samples and remove excess stain. The number of the cells migrate through the membrane was counted using an inverted microscope (Nikon, Japan, magnification × 200).

### Luciferase assay

For the luciferase reporter assay, the WT or MUT NEAT1 constructs were subcloned into the pGL3 basic vector (Promega, Madison, WI, USA). The cells were cotransfected with 5 µl miR-101 mimics and miR-101 NC and with 5 µg WT-NEAT1 or MUT-NEAT1, followed by incubation for 48 h. The luciferase activity was assayed using a Dual-Luciferase® reporter assay system (Promega, Madison, WI, USA), and firefly luciferase activity was normalized to the Renilla activity.

### Orthotopic bladder cancer model

Female BALB/c-nu mice (4–6 weeks of age) were purchased from Nanjing Institute of Biomedicine (Nanjing, China; licence No.: SCXK(Su)2015-0001). Mice were randomly divided into 3 groups: the CON (n = 10), NC (n = 10) and NEAT1 groups (n = 10). After mice were anaesthetized successfully with 1% pentobarbital sodium (45 mg/kg), the lower abdomen of the mice was pressed to empty the bladder. A catheter (no. 7 venous indwelling needle) was inserted into the bladder and flushed with PBS. After treatment with 100 μl of 0.1 g/L HCl, 100 μL of 0.1 g/L KOH and phosphoric acid buffer, the external orifice of the urethra was ligated for 30 min. Then, Hank's balanced salt solution (HBSS) containing 1.5 × 10^6^ cancer cells per 150 μL (T24/5637) was injected into the bladder and retained for 1 h. Four weeks after tumour cell inoculation, the mice were sacrificed to collect the tissues for subsequent experiments.

### Haematoxylin and eosin staining

The tissue samples were fixed in 4% paraformaldehyde for more than 24 h and then embedded in paraffin. After cutting 4 μm-thick sections, the samples were subjected to routine steps of desiccation, haematoxylin–eosin staining, and clearing. The images were imaged under a microscope (magnification, × 200).

### Immunohistochemistry

The tissue sections were blocked with 5% bovine serum albumin for 20 min and incubate with an anti-VEGF-C antibody (1:1000; ab9546, Abcam, USA) overnight at 4 °C. Then, the tissue samples were incubated with a secondary antibody at 37 °C for 30 min. The sections were then stained with DAB (R&D Systems, Minneapolis, MN, USA). The staining results and images were assessed using a light microscope (magnification, × 200).

### Statistical analysis

Statistical analysis was performed using SPSS 22.0 statistical software (SPSS Inc., Chicago, IL, USA). In vitro experiments were repeated in triplicate. The data are presented as the mean ± SD. The differences between the groups were determined using Student’s t test and one-way analysis of variance (ANOVA). One-way ANOVA followed by Tukey's post-hoc test was used to determine significance. Kaplan–Meier analysis with the log-rank test was used to evaluate the recurrence-free survival of patients. The *p* values less than 0.05 were considered statistically significant.

## Results

### The expression of NEAT1, miR-101 and VEGF-C in BC patients

**Fig. 1:**
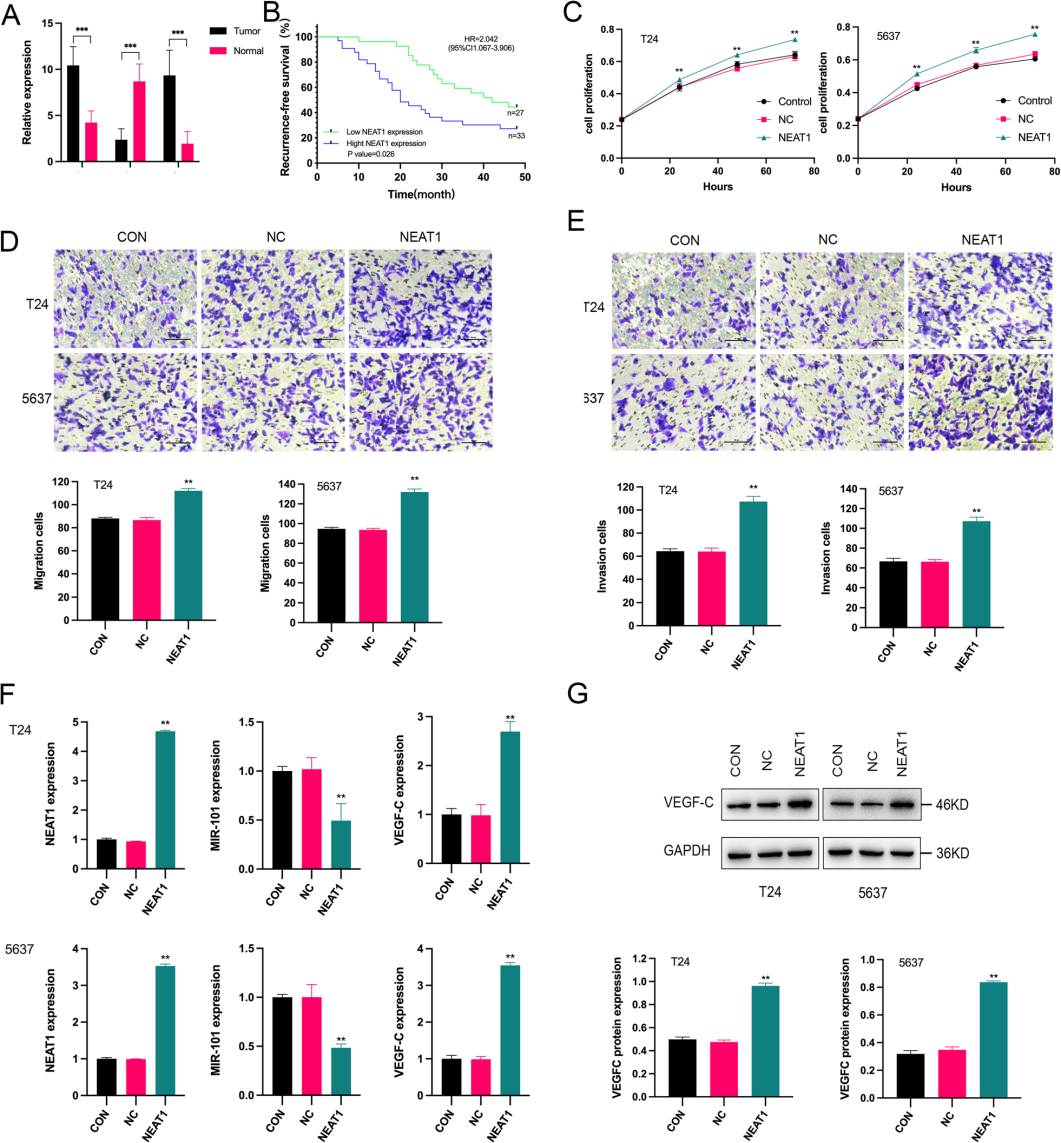
The expression of NEAT1, miR-101 and VEGF-C in BC and the effects on BC cells. **A** The expression of NEAT1 and VEGF-C in the tumour tissues was significantly higher than that in the normal tissues. The expression of miR-101 in tumour tissues was significantly lower than that in the normal tissues. ***p < 0.001 tumour tissues versus normal tissues. **B** The results of Kaplan–Meier analysis revealed that BC patients with high NEAT1 expression presented poor recurrence-free survival. *p* = 0.026 high NEAT1 expression group versus low NEAT1 expression group (HR = 2.042, 95% CI 1.067–3.906). **C** After 24 h, the proliferation of the cells in the NEAT1 group was significantly higher than that in the CON and NC groups in both T24 and 5637 cells. **p < 0.01 NEAT1 group versus CON and NC groups. **D**, **E** The qualitative results of the migration and invasion assays showed that the migration and invasion of the cells in the NEAT1 group were significantly higher than those in the CON and NC groups, both in T24 and 5637 cells. **p < 0.01 NEAT1 group versus CON and NC groups. **F** The mRNA expression of NEAT1 and VEGF-C was significantly increased after NEAT1 transfection, both in T24 and 5637 cells. **p < 0.01 NEAT1 group versus CON and NC groups. The mRNA expression of miR-101 was significantly decreased after NEAT1 transfection. **p < 0.01 NEAT1 group versus CON and NC groups. **G** VEGF-C protein expression was significantly increased after NEAT1 transfection, both in T24 and 5637 cells (Additional file 1: Figure S1). **p < 0.01 NEAT1 group versus CON and NC groups

To investigate the effect of NEAT1, miR-101 and VEGF-C in BC, the expression of NEAT1, miR-101 and VEGF-C in 60 BC tissue samples was determined using qRT–PCR. NEAT1 and VEGF-C were expressed at high levels in the tumour tissues compared to that in adjacent normal tissues, and miR-101 was significantly downregulated (Fig. 1A). We classified BC patients according to the average NEAT1 expression level (10.44 ± 2.02). Patients with NEAT1 expression higher than 10.44 ± 2.02 were assigned to the high NEAT1 expression group (n = 35), and patients with NEAT1 expression lower than 10.44 ± 2.02 were assigned to the low NEAT1 expression group (n = 25). The clinical data indicate the lack of significant difference between the high and low NEAT1 expression groups with regards to age, tumour diameter and tumour number. However, NEAT1 expression was significantly associated with advanced pathological stage and grade (Table 1). Moreover, the results of Kaplan–Meier analysis revealed that BC patients with high NEAT1 expression levels presented with poorer recurrence-free survival than those with low NEAT1 expression levels (Fig. 1B). Therefore, we demonstrated that NEAT1 was upregulated in BC patients and correlated with a poor prognosis.

**Table 1:** Comparison of characteristics of the BC patients in high and low NEAT1 expression group

Parameter	N	Low NEAT1 (N = 27)	High NEAT1 (N = 33)	*p* value
*Age* (year)				
≥ 60	46	22	24	0.425
< 60	14	5	9	
*Tumor diameter* (cm)				0.549
< 3	33	16	17	
≥ 3	27	11	16	
*Tumor number (n)*				0.271
Single	40	20	20	
Multiple	20	7	13	
*Pathological stage*				0.04
T1	29	17	12	
T2–T4	31	10	21	
*Pathological grade*				0.001
G1	26	18	8	
G2–G3	34	9	25	

### Effect of NEAT1 expression on the malignant cellular phenotypes of BC cells

To further study the roles of NEAT1 in BC, T24 and 5637 BC cells were used for the experiments. NEAT1 expression was induced in the T24 and 5637 BC cell lines. The results of the MTT assay revealed that the proliferation of the cells in the NEAT1 group was greatly promoted compared with that in the CON and NC groups in a time-dependent manner (Fig. 1C). The results of the migration and invasion assays showed that upregulation of NEAT1 enhanced the migration and invasion of T24 and 5637 cells (Fig. 1D, E). These results suggested that NEAT1 was involved in the progression of BC.

### Effect of NEAT1 on the expression of miR-101 and VEGF-C in BC cells

After the introduction of NEAT1, the transfection efficacy was confirmed by the upregulation of NEAT1 using qRT–PCR (Fig. 1F). The data showed a significant promotion of the expression of VEGF-C in the NEAT1 group and a significant inhibition of the expression of miR-101 compared with those in the NC and CON groups (Fig. 1F). The protein expression of VEGF-C was in agreement with the mRNA expression (Fig. 1G). No notable differences were detected between the NC and CON groups.

### NEAT1 negatively regulated the expression of miR-101 in BC cells

**Fig. 2:**
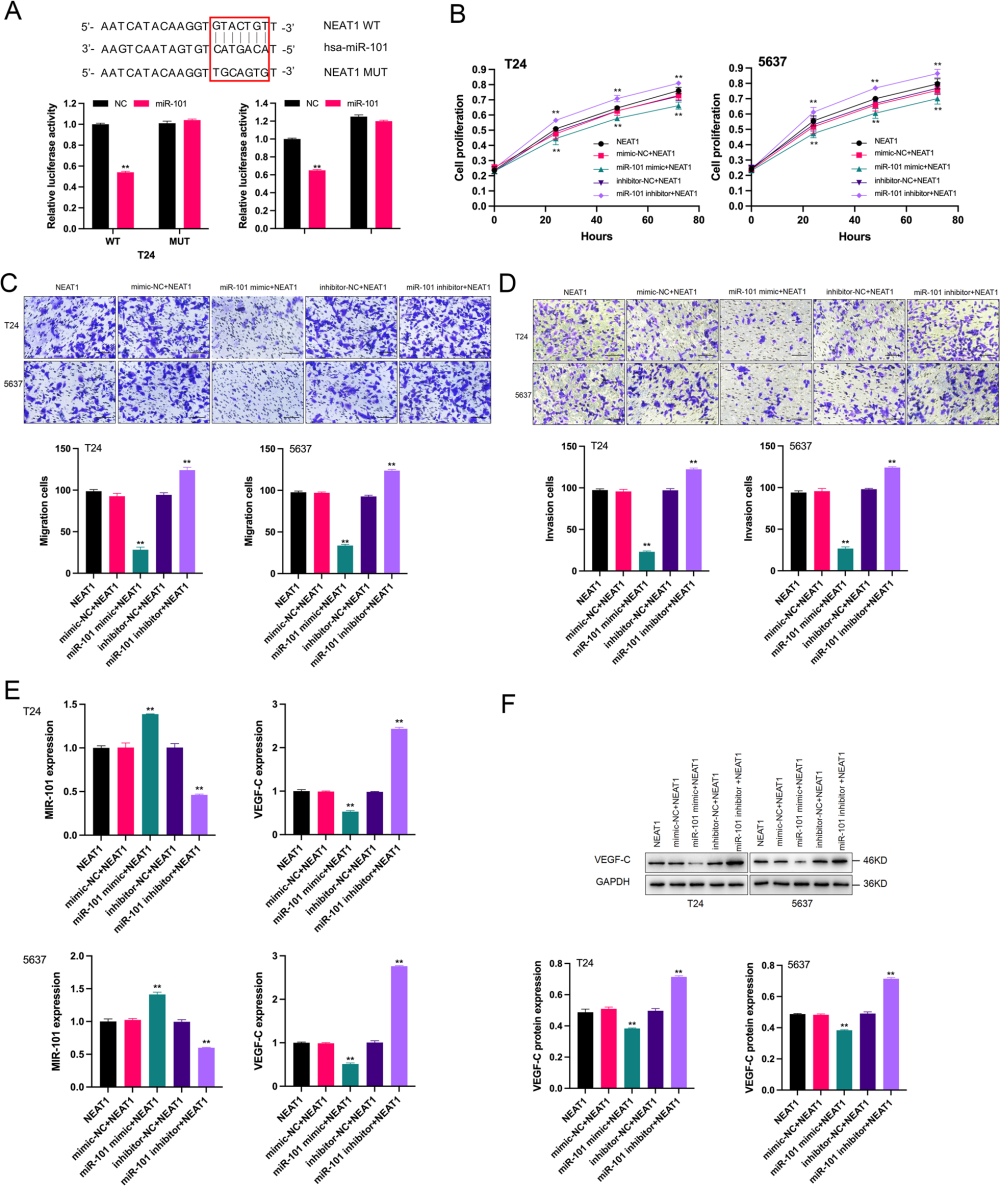
Effects of the NEAT1/miR-101 pathway on BC cells. **A** The putative binding sites of NEAT1 with miR-101 are listed. The results of the luciferase assay indicated that overexpressed miR-101 significantly decreased the activity of the wild-type NEAT1 3′-UTR reporter but did not influence the activity of the mutant NEAT1 3′-UTR reporter. **p < 0.01 miR-101 group versus NC group. **B** After 24 h, the proliferation of the cells with enforced NEAT1 expression and a miR-101 inhibitor was significantly higher than that in the NEAT1 group in both T24 and 5637 cells. **p < 0.01 miR-101 inhibitor + NEAT1 group versus NEAT1 group. The proliferation of the cells with enforced NEAT1 expression and a miR-101 mimic was significantly lower than that in the NEAT1 group in both T24 and 5637 cells. **p < 0.01 miR-101 mimic + NEAT1 group versus NEAT1 group. **C**, **D** The quantitative results of the migration and invasion assays showed that the migration and invasion of the cells with enforced NEAT1 expression and a miR-101 inhibitor were significantly higher than those in the NEAT1 group both in T24 and 5637 cells. **p < 0.01 miR-101 inhibitor + NEAT1 group versus NEAT1 group. The migration and invasion of the cells with enforced NEAT1 expression and a miR-101 mimic were significantly lower than those in the NEAT1 group in both T24 and 5637 cells. **p < 0.01 miR-101 mimic + NEAT1 group versus NEAT1 group. **E** The expression of miR-101 mRNA was significantly increased after transfection with NEAT1 and a miR-101 mimic and decreased after transfection with NEAT1 and a miR-101 inhibitor. **p < 0.01 miR-101 mimic + NEAT1/miR-101 inhibitor + NEAT1 group versus NEAT1 group. The expression of VEGF-C mRNA was significantly decreased after transfection with NEAT1 and a miR-101 mimic and increased after transfection with NEAT1 and a miR-101 inhibitor. **p < 0.01 miR-101 mimic + NEAT1/miR-101 inhibitor + NEAT1 group versus NEAT1 group. **F.** The VEGF-C levels of the cells were significantly decreased after transfection with NEAT1 and a miR-101 mimic and increased after transfection with NEAT1 and a miR-101 inhibitor (Additional file 2: Figure S2). **p < 0.01 miR-101 mimic + NEAT1/miR-101 inhibitor + NEAT1 group versus NEAT1 group

The data of qRT–PCR confirmed that the expression of miR-101 was clearly downregulated in the NEAT1 group. We speculated that miR-101 may be a target gene of NEAT1. To establish direct targeting of NEAT1 by miR-101, the wild-type targeting sequence of NEAT1 (WT-NEAT1) and mutated vectors (MUT-NEAT1) were constructed. The luciferase reporter plasmid containing WT NEAT1 and miR-101 mimic were cotransfected, and a decrease in the reporter activity was demonstrated; however, the activity of the MUT NEAT1 construct was unaltered under the same conditions (Fig. 2A). These results indicated that miR-101 was a target of NEAT1 and that NEAT1 negatively regulated miR-101 levels.

### NEAT1 modulated VEGF-C expression by sponging miR-101 to promote the malignant phenotypes of BC cells

**Fig. 3:**
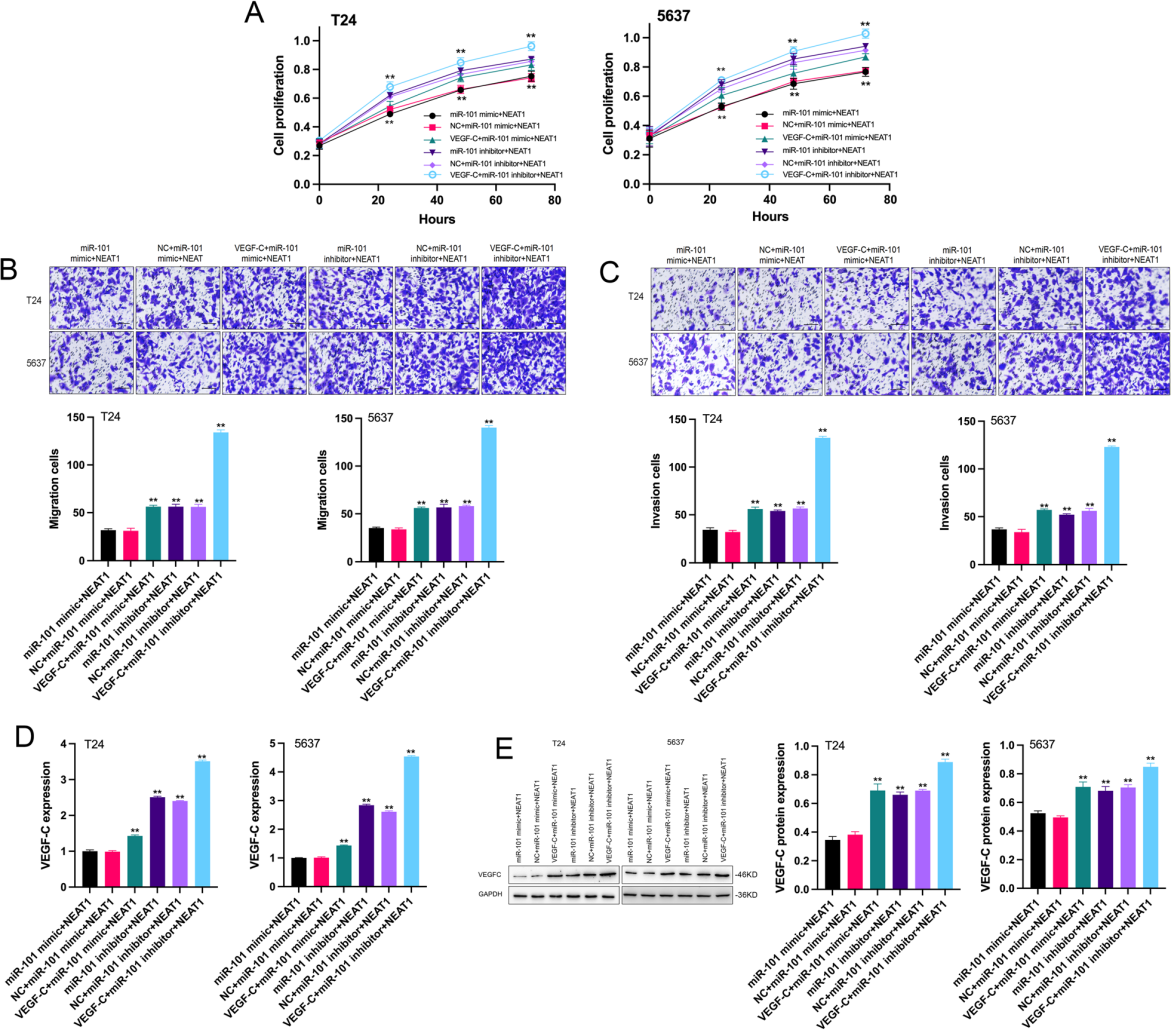
Effects of the NEAT1/miR-101/VEGF-C pathway on BC cells. **A** After 24 h, the proliferation of the cells with enforced VEGF-C, NEAT1 and miR-101 mimic/inhibitor expression was significantly higher than that in the miR-101 mimic + NEAT1 group both in T24 and 5637 cells. **p < 0.01 VEGF-C + miR-101 mimic/inhibitor + NEAT1 group versus miR-101 mimic + NEAT1 group. The proliferation of the cells with enforced NEAT1 and miR-101 inhibitor expression was also significantly higher than that in the miR-101 mimic + NEAT1 group both in T24 and 5637 cells. **p < 0.01 miR-101 inhibitor + NEAT1 group versus miR-101 mimic + NEAT1 group. **B**, **C** The quantitative results of the migration and invasion assay showed that the migration and invasion of the cells with enforced VEGF-C, NEAT1 and miR-101 mimic/inhibitor expression were significantly higher than those in the miR-101 mimic + NEAT1 group both in T24 and 5637 cells. **p < 0.01 VEGF-C + miR-101 mimic/inhibitor + NEAT1 group versus miR-101 mimic + NEAT1 group. The migration and invasion of the cells with enforced NEAT1 and miR-101 inhibitor expression were also significantly higher than those in the miR-101 mimic + NEAT1 group both in T24 and 5637 cells. **p < 0.01 miR-101 inhibitor + NEAT1 group versus miR-101 mimic + NEAT1 group. **D**, **E** The mRNA and protein expression of VEGF-C were all significantly increased in the VEGF-C + miR-101 mimic + NEAT1, miR-101 inhibitor + NEAT1 and VEGF-C + miR-101 inhibitor + NEAT1 groups (Additional file 3: Figure S3). **p < 0.01 VEGF-C + miR-101 mimic + NEAT1, miR-101 inhibitor + NEAT1 and VEGF-C + miR-101 inhibitor + NEAT1 groups versus miR-101 mimic + NEAT1 group

Considering the relationship between NEAT1, miR-101 and VEGF-C revealed by the data of the present study, we hypothesised that miR-101 and VEGF-C mediate the regulation of BC progression by NEAT1. As shown in Fig. 2B–D, contrary to the effects of upregulated NEAT1, the miR-101 mimic inhibited the proliferation, migration and invasion of BC cells, which were rescued by a miR-101 inhibitor. Correspondingly, VEGF-C levels were downregulated by overexpressed miR-101 in T24 and 5637 cells (Fig. 2E, F). However, miR-101 deletion prominently facilitated the expression of VEGF-C (Fig. 2E, F). This result indicated that miR-101 repressed the mRNA and protein levels of VEGF-C in BC cells. Next, we analysed the effects of VEGF-C on NEAT1-mediated promotion in BC cells. First, the results of functional analysis suggested that the introduction of VEGF-C enhanced the positive role of NEAT1 in the proliferation, migration and invasion of BC cells (Fig. 3A–C). Second, qRT–PCR and western blot assays were used to detect the overexpression efficiency of VEGF-C in BC cells (Fig. 3D, E). The data revealed that VEGF-C levels were significantly upregulated in the VEGF-C + miR-101 mimic + NEAT1, miR-101 inhibitor + NEAT1, and VEGF-C + miR-101 inhibitor + NEAT1 groups compared with that in the miR-101 mimic + NEAT1 group. Overall, these results showed that NEAT1 acted as a sponge of miR-101 to regulate VEGF-C to influence the progression of BC.

### NEAT1 promoted tumour growth and metastasis of BC

**Fig. 4:**
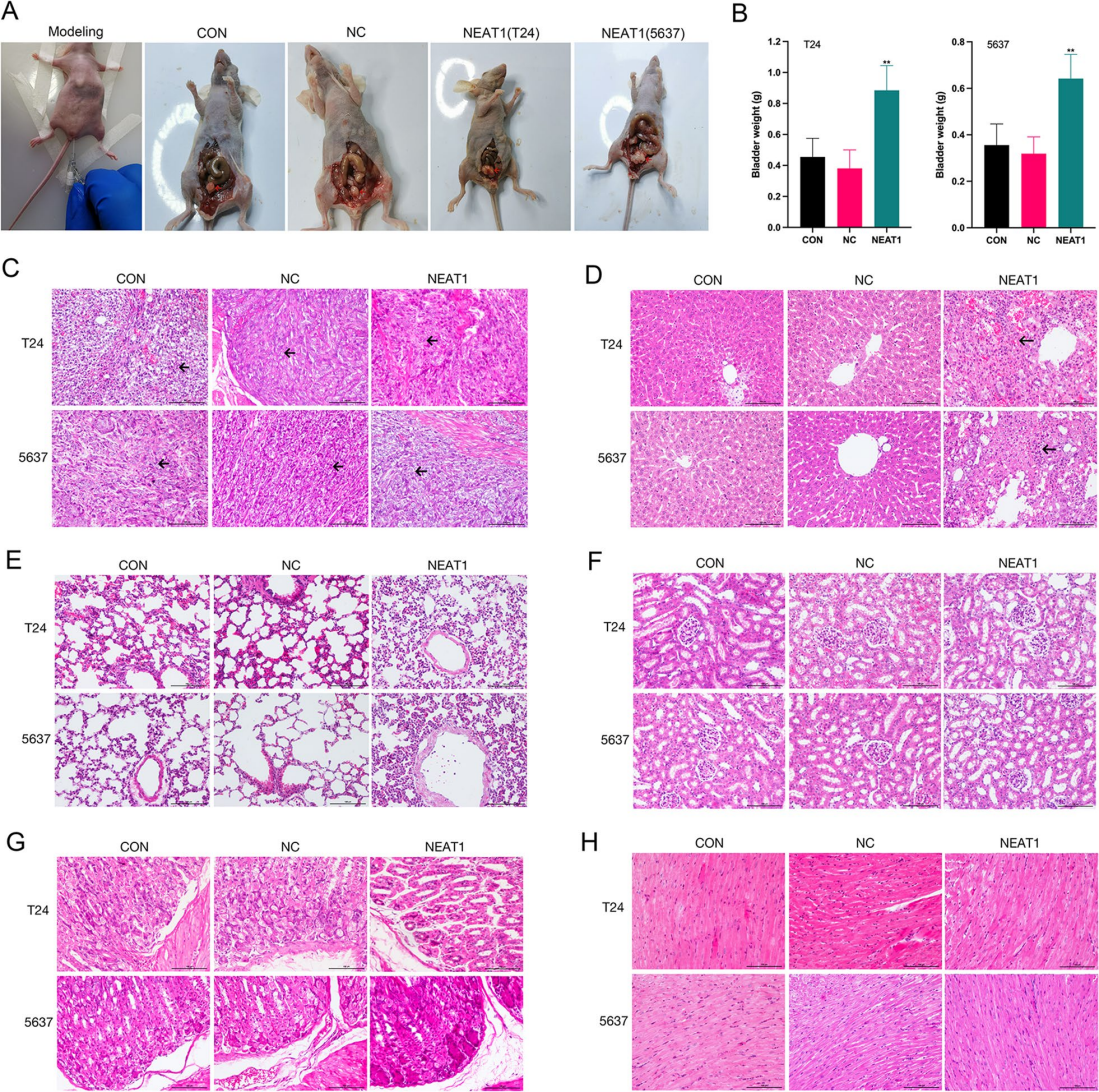
The effects of enforced NEAT1 on tumour growth and metastasis of BC. **A** The tumour implant was established as an orthotopic bladder cancer model, leading to tumorigenicity in vivo (the bladder model is marked by the red arrow). **B** The bladder weight of mice was significantly increased in the NEAT1 group; **p < 0.05 NEAT1 group versus CON and NC groups. **C–H** Histopathological cell morphology of the bladder, liver, lung, kidney, stomach and heart tissues was examined by H&E staining (magnification, × 200). Bladder cancer cells showed high-grade dysplasia, disorder, hyperchromatic nuclei and nuclear division in bladder tissues in all groups (the cancer cell is marked by the black arrow). Liver metastasis occurred only in the NEAT1 group, as indicated by necrosis of large numbers of hepatocytes and infiltration of cancer cells (the metastatic foci is marked by the black arrow). No metastatic lesions were detected in the lung, kidney, stomach and heart, in the three groups

**Fig. 5:**
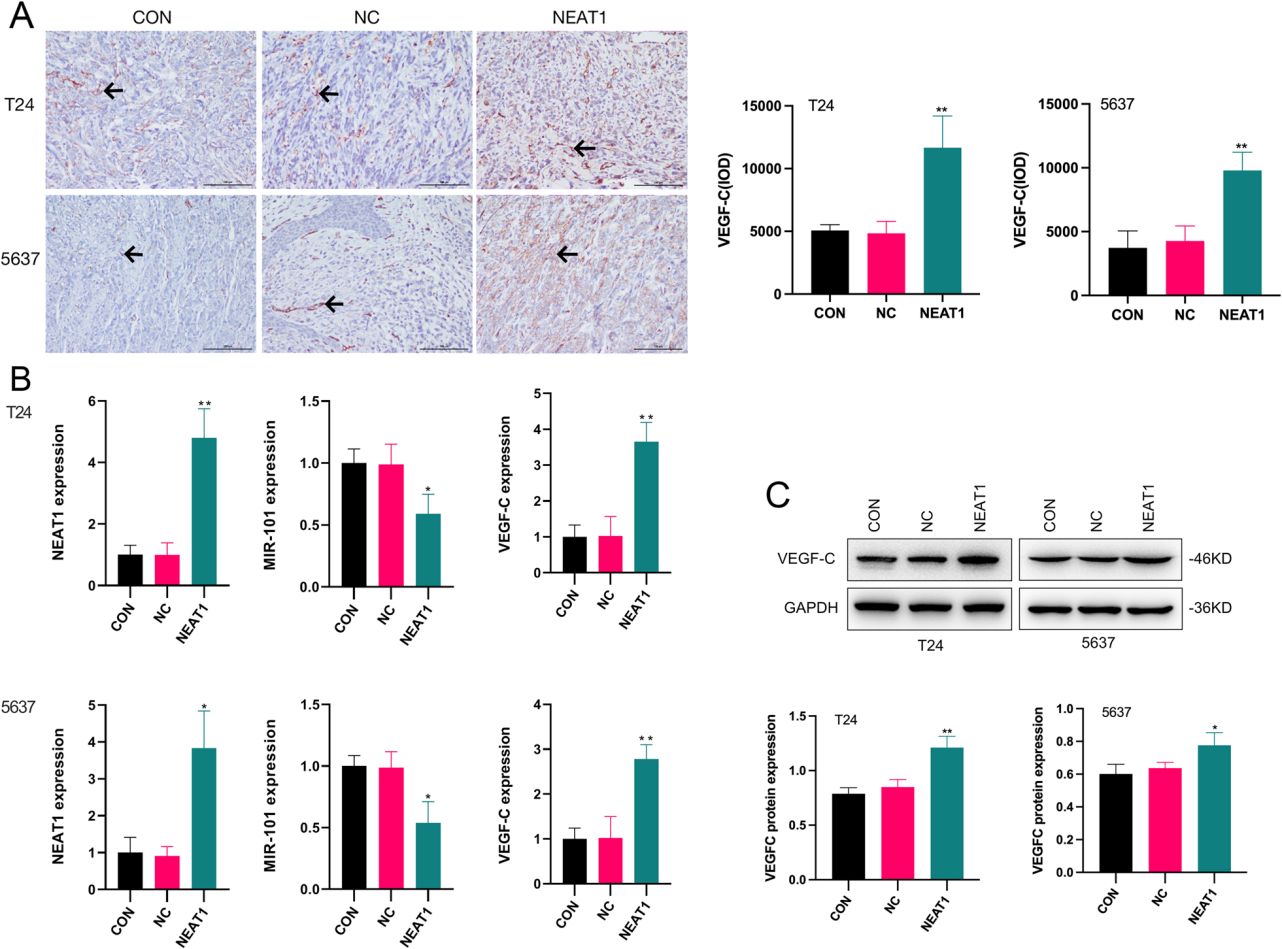
The expression of NEAT1, miR-101 and VEGF-C in an orthotopic bladder cancer model. **A** The results of immunohistochemical staining showed that VEGF-C expression was significantly increased in the NEAT1 group (the immunohistochemical positive cell is marked by the black arrow), **p < 0.01. NEAT1 group versus CON and NC groups (magnification × 200). **B** The expression of NEAT1 and VEGF-C mRNAs were significantly increased after NEAT1 transfection. *p < 0.05, **p < 0.01 NEAT1 group versus CON and NC groups. The expression of miR-101 mRNA was significantly decreased after NEAT1 transfection. *p < 0.05, **p < 0.01 NEAT1 group versus CON and NC groups. **C** The expression of VEGF-C protein was significantly increased after NEAT1 transfection (Additional file 4: Figure S4). *p < 0.05, **p < 0.01 NEAT1 group versus CON and NC groups

To further confirm the effect of NEAT1 on tumour growth, we generated an orthotopic BC model in vivo. The model was successfully established and validated by the detection of a rigid mass in the lower abdomen (Fig. 4A). In T24 cells group, the tumorigenesis rates were 70%, 80% and 80% in CON, NC and NEAT1 group, respectively. In 5637 cells group, the tumorigenesis rates were 70%, 80% and 70% in CON, NC and NEAT1 group, respectively. There was no difference between the two kinds of cells. The differences in the bladder weight between the groups were statistically significant. Comparison with the CON and NC groups indicated that the bladder weight in the NEAT1 group was significantly increased (Fig. 4B). This result indicated that NEAT1 promoted the tumour growth of BC in vivo. The orthotopic tumour model was further confirmed by H&E imaging. In all three groups, epithelial cells of the bladder tissues manifested high-grade dysplasia and variable sizes. Morphological observations indicated that the cells were disarranged, with hyperchromatic nuclei and visible nuclear division (Fig. 4C–H). This result demonstrated that tumour lesions were successfully induced in all three groups. Notably, no metastatic lesions were detected in the lung, heart, kidney or stomach in any of the three groups; however, metastatic lesions were detected in the liver in the NEAT1 group. In T24 cells group, the rates of liver metastasis were 0%, 0%, and 40% in CON, NC and NEAT1 group, respectively. In 5637 cells group, the tumorigenesis rates were 0%, 0%, and 30% in CON, NC and NEAT1 group, respectively. There was no difference between the two kinds of cells. The results of the immunohistochemistry, qRT–PCR and western blot assays showed that VEGF-C expression was significantly higher in the NEAT1 group and miR-101 expression was significantly suppressed compared with those in the CON and NC groups (Fig. 5). These results indicated that NEAT1 promoted tumour growth and metastasis of BC via the miR-101/VEGF-C axis.

## Discussion

In recent years, microRNAs (miRNAs) and long noncoding RNAs (lncRNAs) have attracted the attention of researchers in the field of cancer biology because of their role in almost every aspect of cellular functions [9]. Importantly, these molecules are involved in tumour initiation and progression. Our previous study demonstrated that the expression of miR-101 was downregulated in BC, which consequently favours tumour progression [10]. Moreover, we demonstrated that VEGF-C protein expression was increased in the BC tissues. Increased VEGF-C expression is associated with a poorer prognosis [11]. MiR-101 was reported to suppress the migration and invasion of hepatocellular carcinoma cells by negatively regulating VEGF-C expression [12]. In the present study, we aimed to determine whether NEAT1 regulates BC progression through the miR-101/VEGF-C axis.

The results of the present study indicated that NEAT1 functioned as an oncogene in BC. The expression of NEAT1 was higher in the BC tissues than that in the normal tissues, and increased NEAT1 expression in the BC tissues was significantly correlated with poorer recurrence-free survival. NEAT1 upregulation may be able to predict disease progression; however, the effect of NEAT1 on the overall survival of BC patients was not explored in the present study due to the lack of long-term follow-up investigation.

The results of the experiments showed that overexpression of NEAT1 significantly promoted the proliferation, migration and invasion of BC cells in vitro. To further elucidate a potential molecular mechanism underlying the oncogenic function of NEAT1 in BC progression, we focused on the associations between NEAT1 and the miR-101/VEGF-C axis. To investigate the correlations of NEAT1 and the miR-101/VEGF-C axis, we detected the expression of these molecules in BC patients and controls, and the data showed that NEAT1 and VEGF-C were both upregulated and miR-101 was downregulated in BC patients compared with those in the control group. In BC patients, NEAT1 was positively correlated with VEGF-C and negatively correlated with miR-101. According to the ceRNA hypothesis, lncRNAs competitively bind certain miRNAs and serve as decoys, thereby restoring the expression of targeted mRNA and resulting in the formation of the lncRNA/miRNA/mRNA pathways [13]. Thus, we proposed that NEAT1 may serve as a ceRNA by targeting miR-101 to upregulate VEGF-C expression, further promoting the onset and development of BC.

MiR-101 downregulation is associated with bladder cancer progression and decreased chemosensitivity [14]. Moreover, miR-101 is a potential biomarker for diagnosis of bladder cancer [15]. Mechanistically, miR-101 has suppressive effects on BC cell proliferation and invasion by inhibiting the expression of its targets FZD4 and c-FOS [16, 17]. In the present study, we confirmed the model of regulation of NEAT1 and miR-101 based on the following results: (1) the results of the Dual-Luciferase® reporter assay revealed that NEAT1 functions via an interaction with miR-101; (2) overexpression of NEAT1 in T24 and 5637 cells significantly decreased miR-101 expression; and (3) a miR-101 mimic significantly reversed NEAT1-mediated stimulatory effects on T24 and 5637 cells, and a miR-101 inhibitor effectively enhanced the effects of NEAT1 on T24 and 5637 cells. These results strongly suggested that NEAT1 expression enhanced the development of BC cells by negatively regulating miR-101.

Vascular endothelial growth factor (VEGF) is a specific heparin-binding growth factor for vascular endothelial cells that can stimulate the formation of new blood vessels [18]. VEGF-C is a lymphangiogenic factor belonging to the VEGF family. BC has a high incidence rate of lymphatic metastasis; hence, VEGF-C has attracted considerable attention, especially in the studies of BC [19]. We aimed to determine whether VEGF-C participates in the NEAT1/miR-101 pathway in BC. To answer this question, we performed qRT–PCR and western blot analysis to measure VEGF-C expression in T24 and 5637 cells after modulation of NEAT1/miR-101 expression. Overexpression of NEAT1 in T24 and 5637 cells significantly decreased miR-101 expression and increased VEGF-C expression. A miR-101 mimic significantly reversed NEAT1-mediated stimulation of T24 and 5637 cells, whereas overexpression of VEGF-C abated the repressive effects of the miR-101 mimic on BC cells. A miR-101 inhibitor significantly enhanced NEAT1-mediated stimulation of T24 and 5637 cells, whereas overexpression of VEGF-C abated the facilitating effects of the miR-101 inhibitor on BC cells. Thus, these data and the results of the experiment on the loss and gain of function of NEAT1/miR-101 in T24 and 5637 cells suggested that we have constructed a novel model of regulation in which abnormal NEAT1 expression modulated BC cell development apparently via the miR-101/VEGF-C pathway. Finally, the in vivo data suggested the stimulatory function of NEAT1 in BC. BC cells with high NEAT1 expression are considerably more likely to present distant metastasis, and this increase in the expression was accompanied by low miR-101 expression and high VEGF-C expression. Therefore, the NEAT1/miR-101/VEGF-C pathway was established.

## Conclusions

In summary, comparison with the normal tissues indicated that NEAT1 was upregulated in BC and was associated with an unfavourable prognosis. In addition, we demonstrated that NEAT1 promoted the malignant development of BC cells. Furthermore, we demonstrated that NEAT1 influenced the development of BC via the modulation of the miR-101/VEGF-C pathway. Thus, the NEAT1/miR-101/VEGF-C pathway may serve as a novel therapeutic target in BC patients. However, only two kinds of the cell lines were used in the present study. Additional research is needed to further explore the mechanism by which NEAT1 is involved in the development of bladder cancer.

## Supplementary Information

**Additional file 1.** Original images of western blots for Fig. 1G.

**Additional file 2.** Original images of western blots for Fig. 2F.

**Additional file 3.** Original images of western blots for Fig. 3E.

**Additional file 4.** Original images of western blots for Fig. 5C.

## Data Availability

The datasets used and/or analysed in the current study y are available from the corresponding author on reasonable request.
