# Supplementary figures and images for: NEAT1 promotes the malignant development of bladder cancer by regulating the miR-101/VEGF-C pathway in vitro and in vivo
Source: BMC Urol. 2022 Nov 25;22:193. doi: 10.1186/s12894-022-01151-z (PMC9700885; doi:10.1186/s12894-022-01151-z)

Supplementary Fig S1

Original western blots for Figure 1G

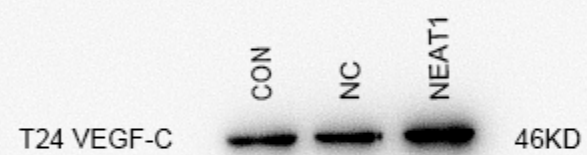

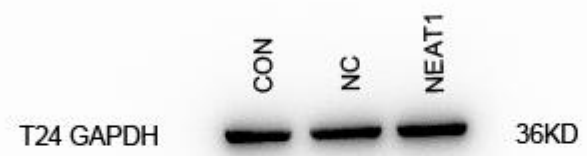

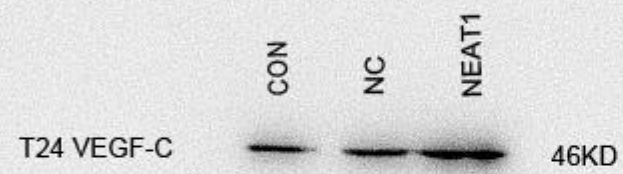

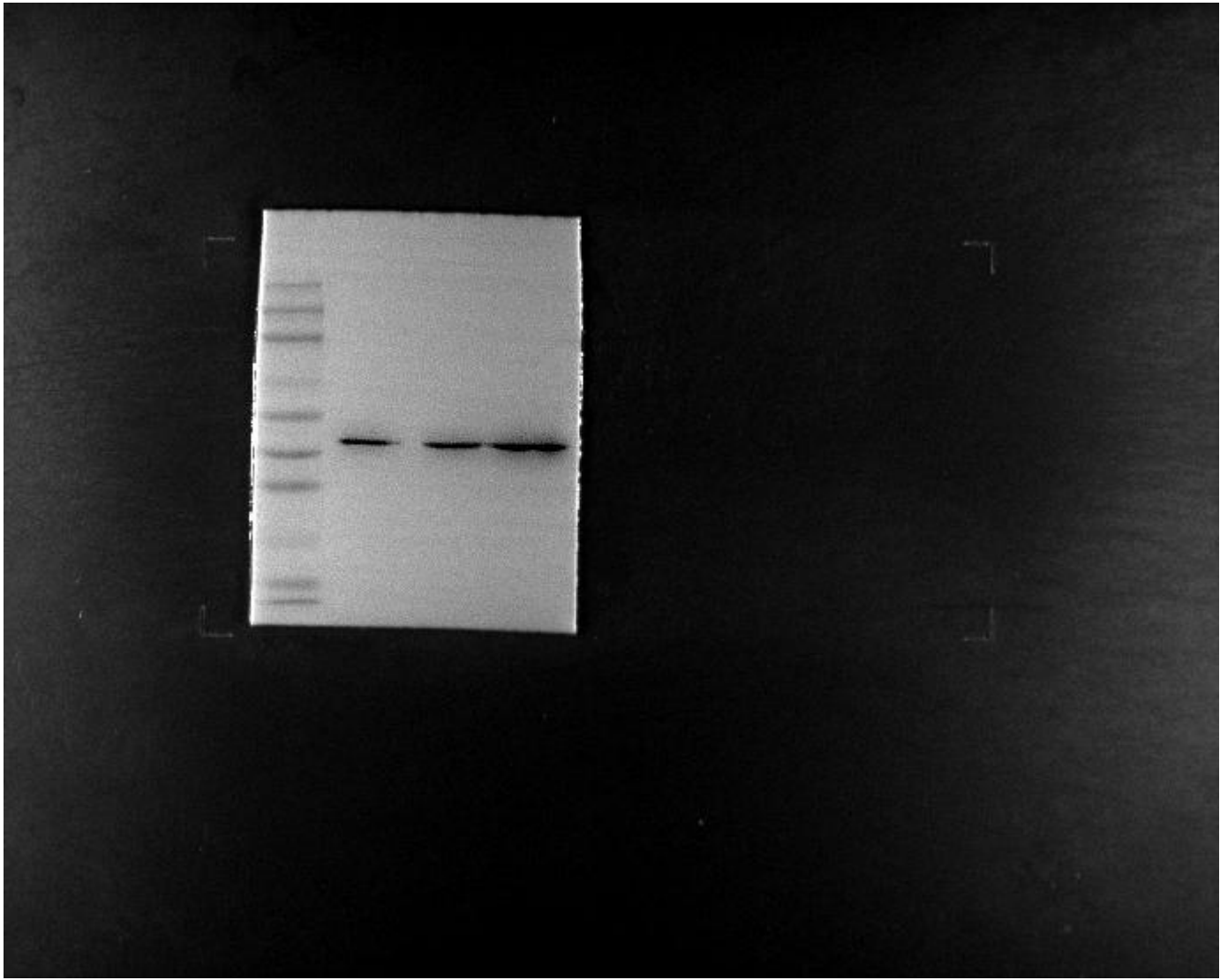

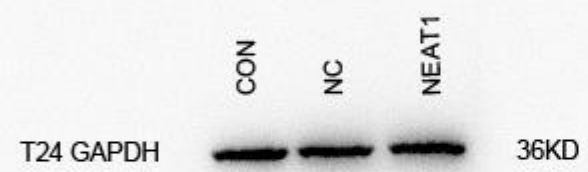

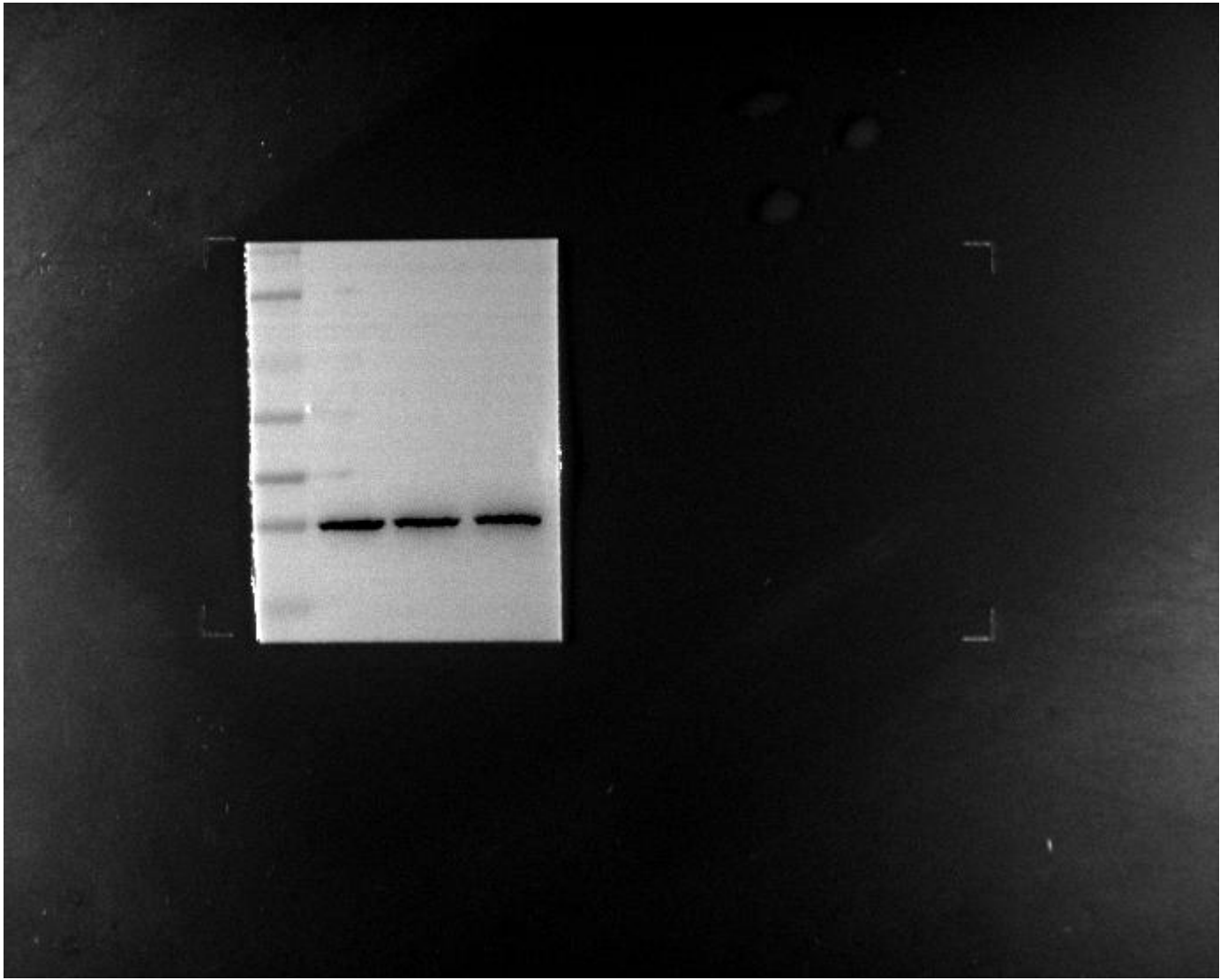

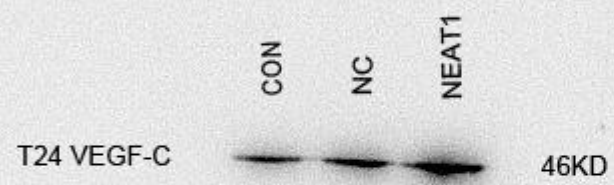

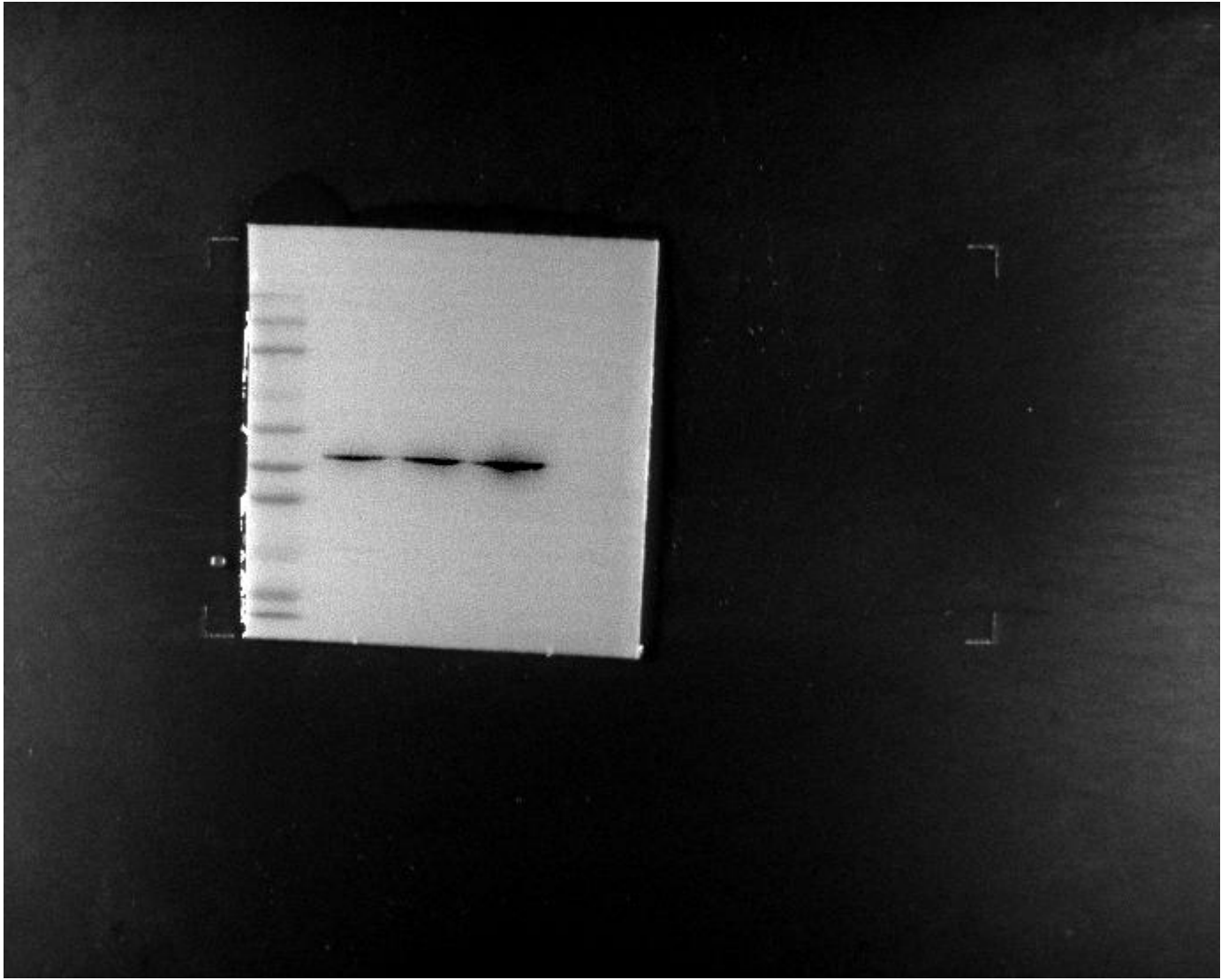

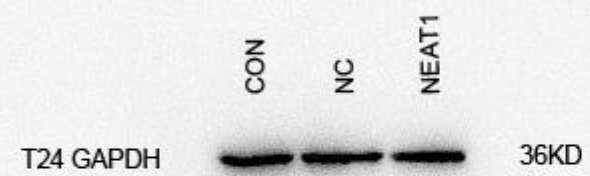

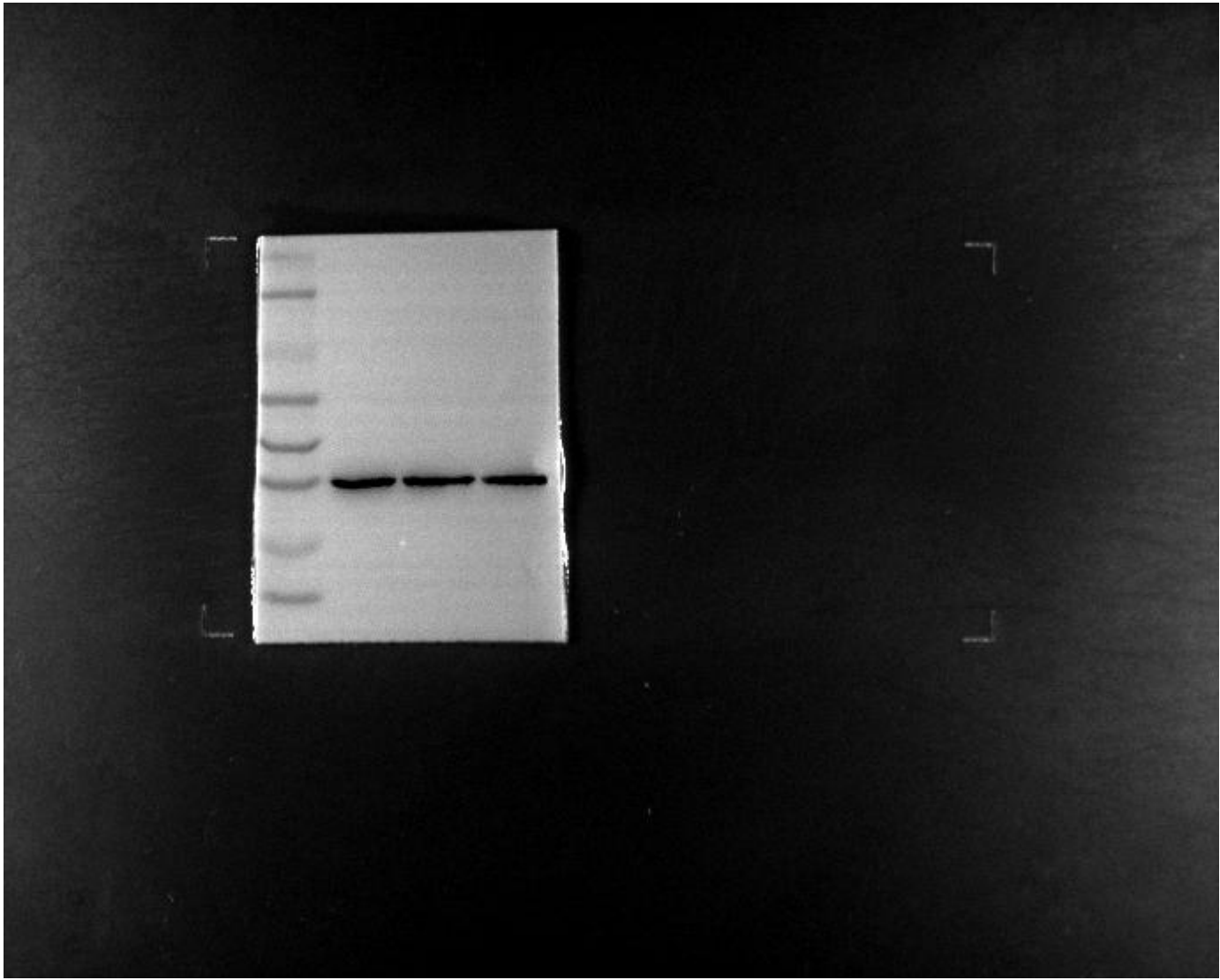

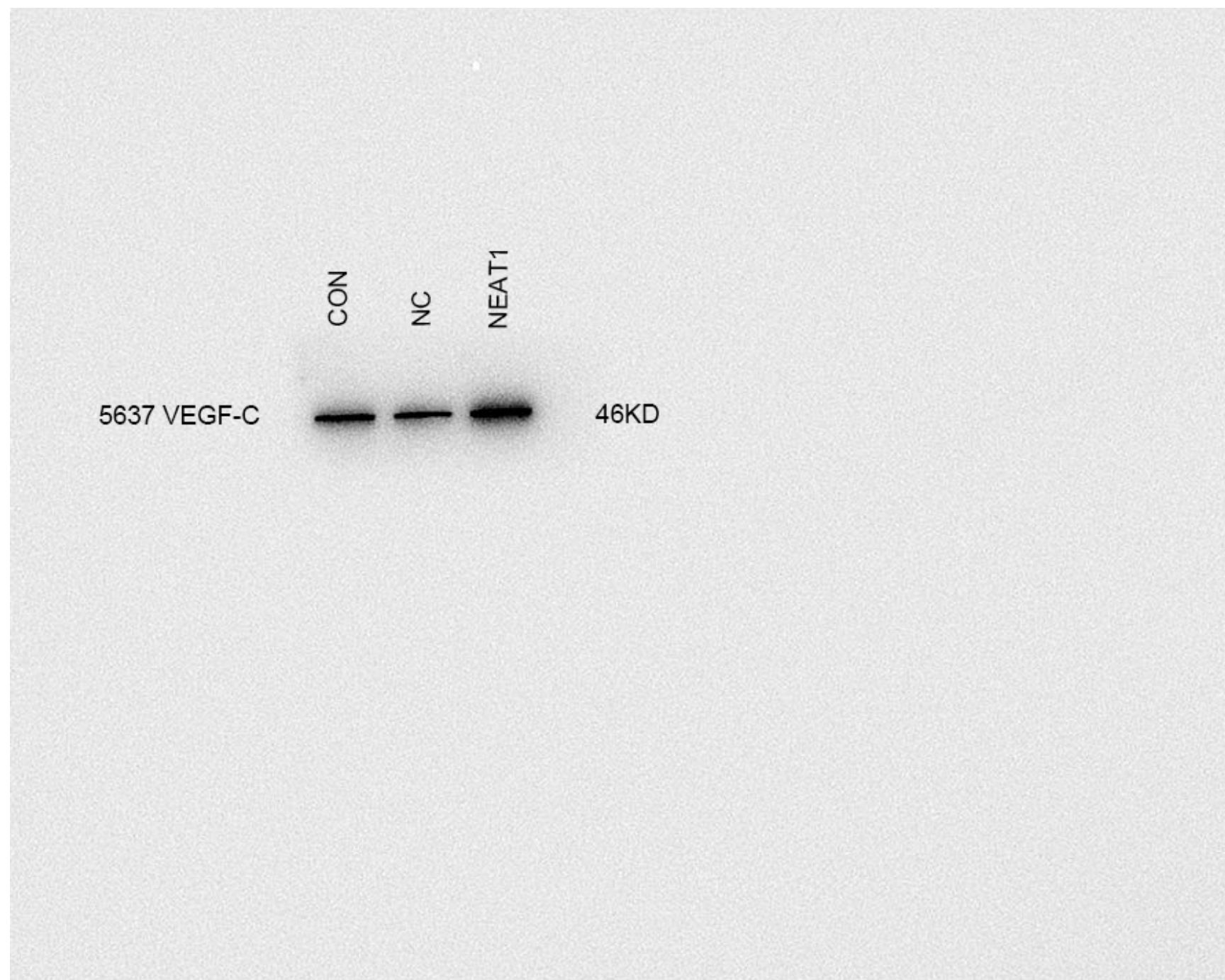

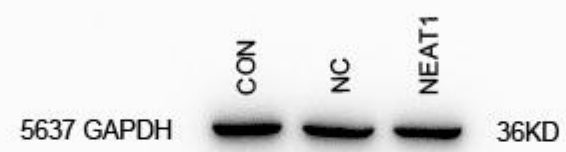

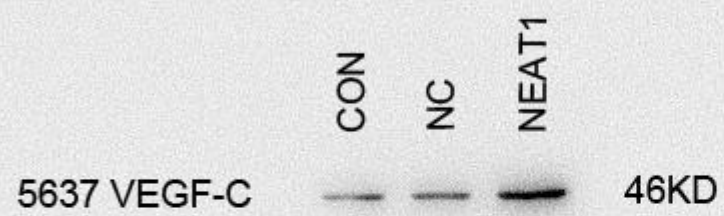

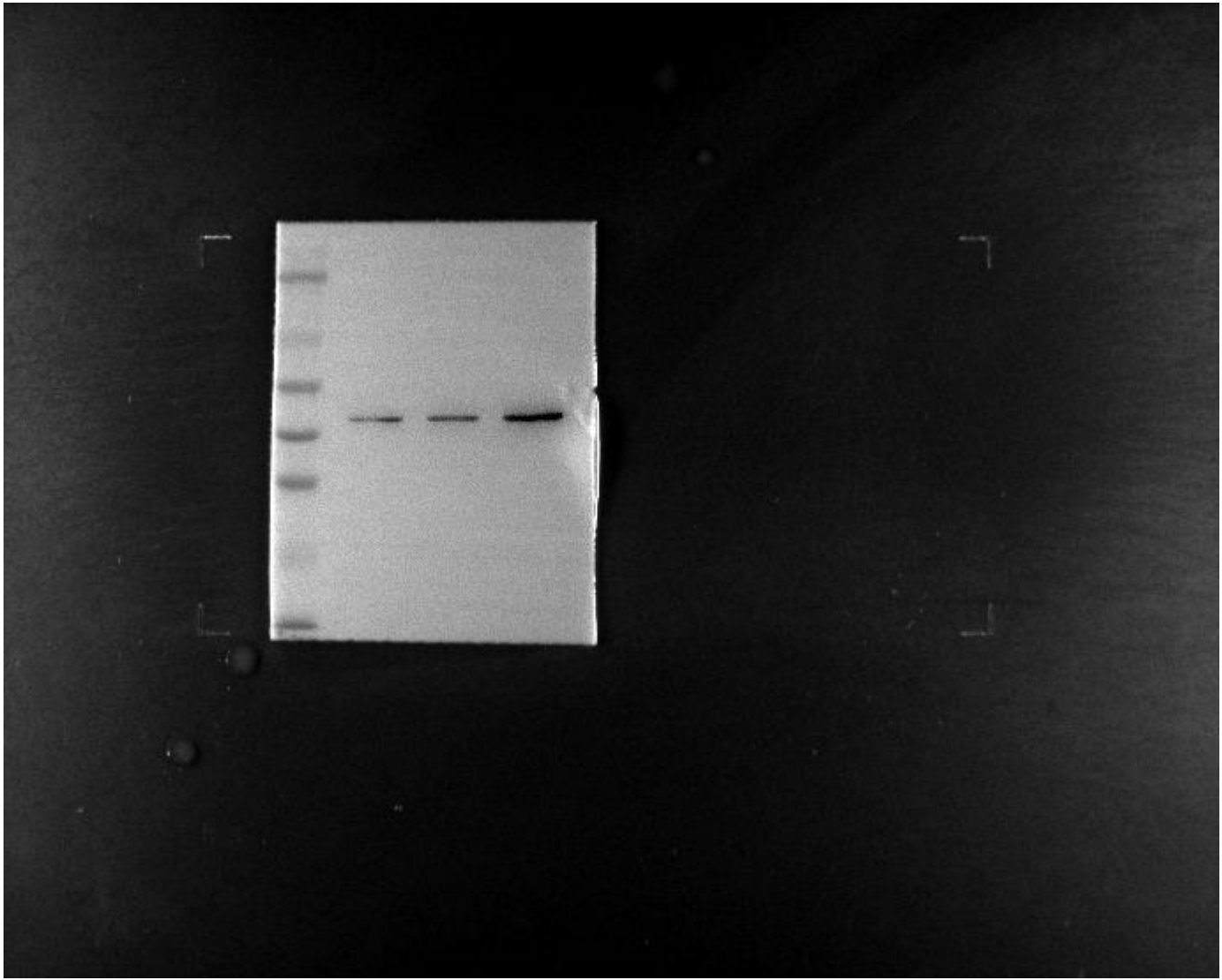

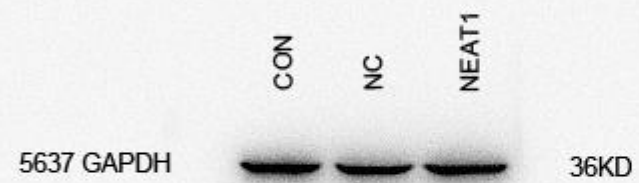

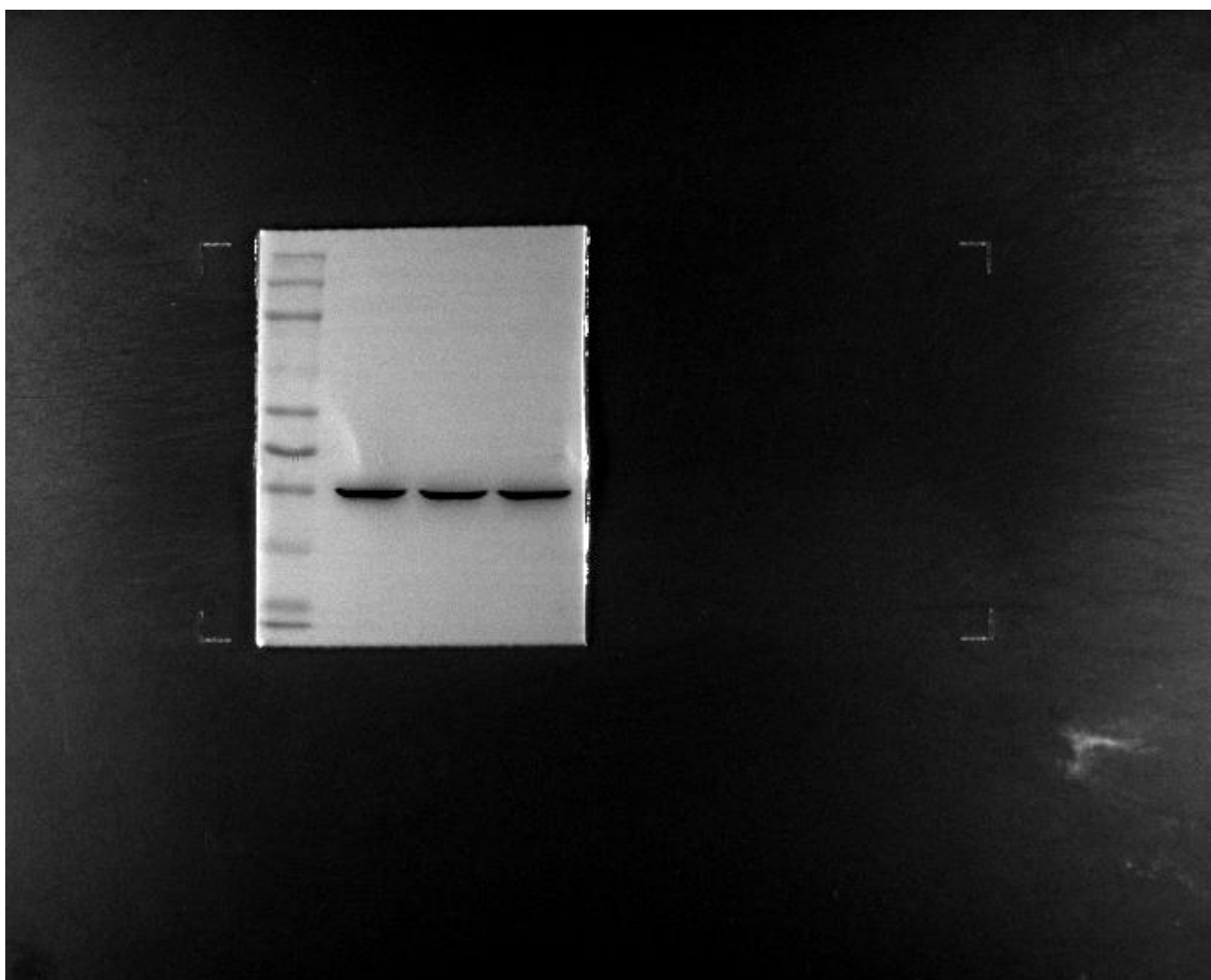

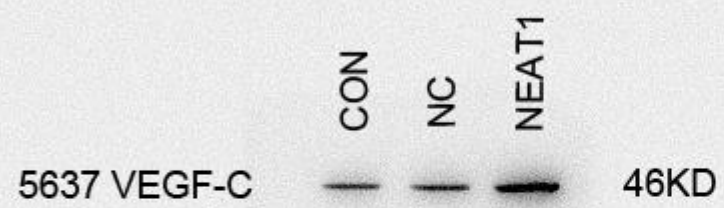

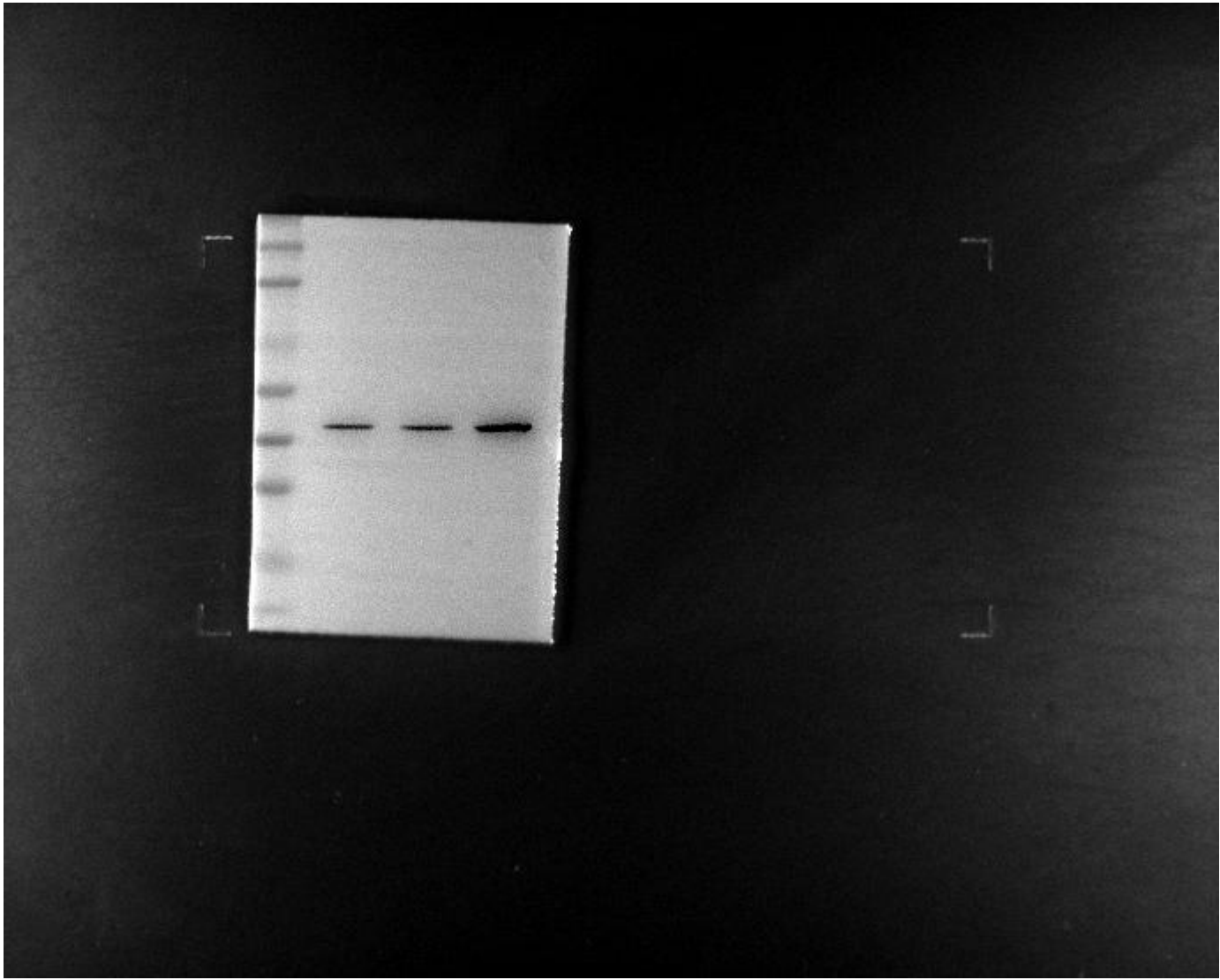

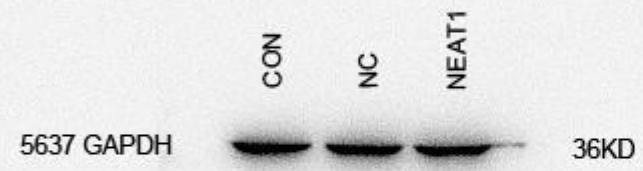

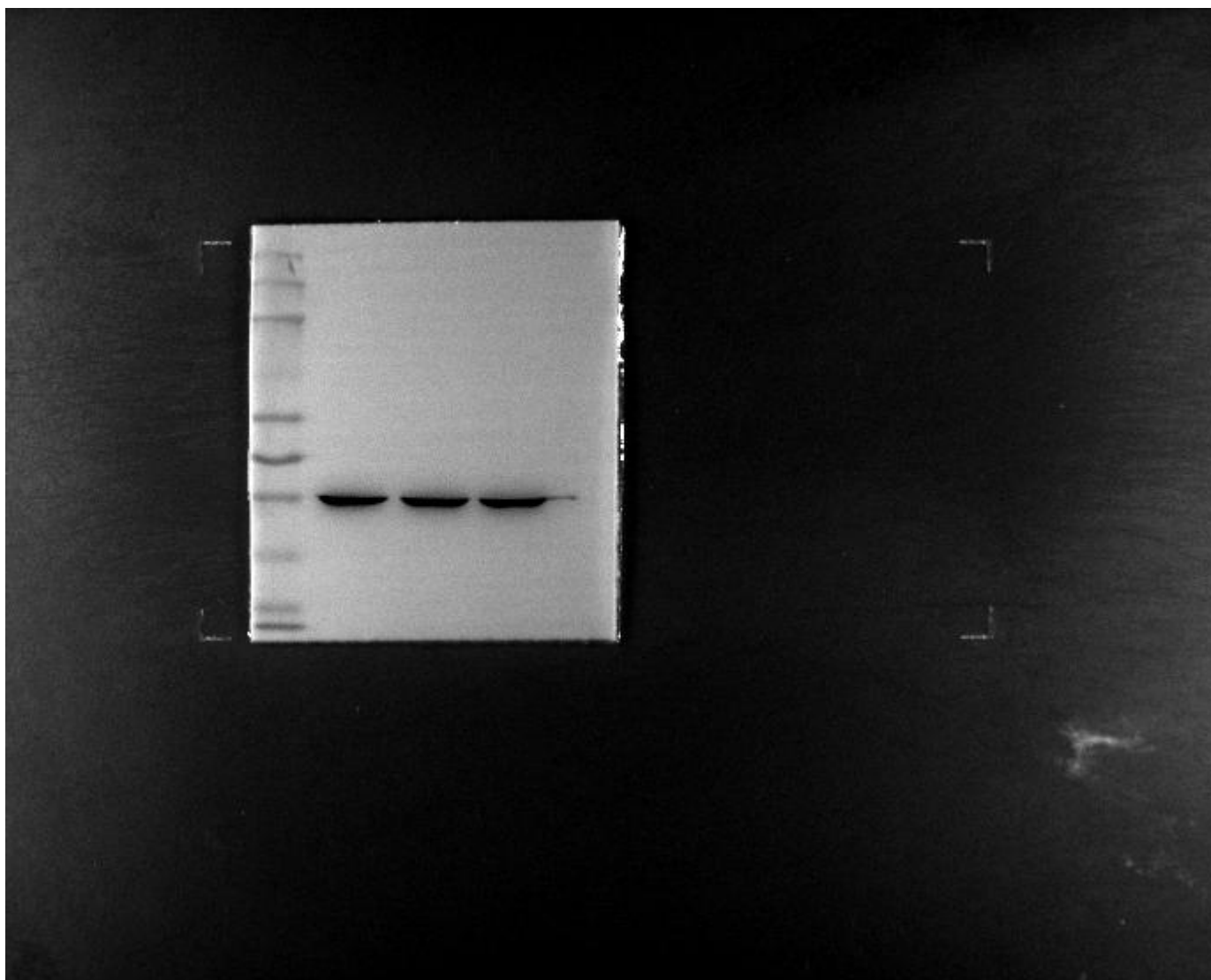

Supplement: Supplementary file 1 — Additional file 1. Original images of western blots for Fig. 1G. [file 12894_2022_1151_MOESM1_ESM.pdf]

# Supplementary Fig S4

Original western blots for Figure 5C

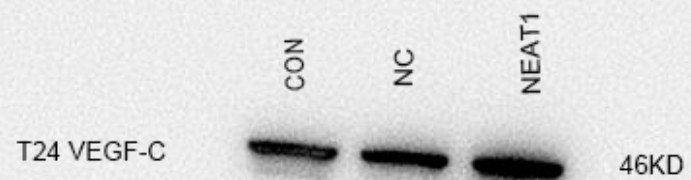

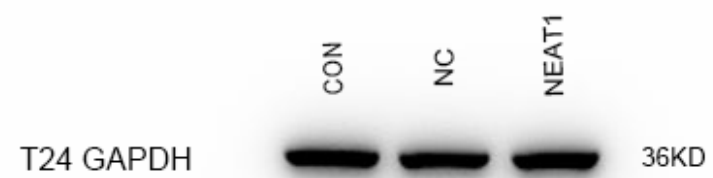

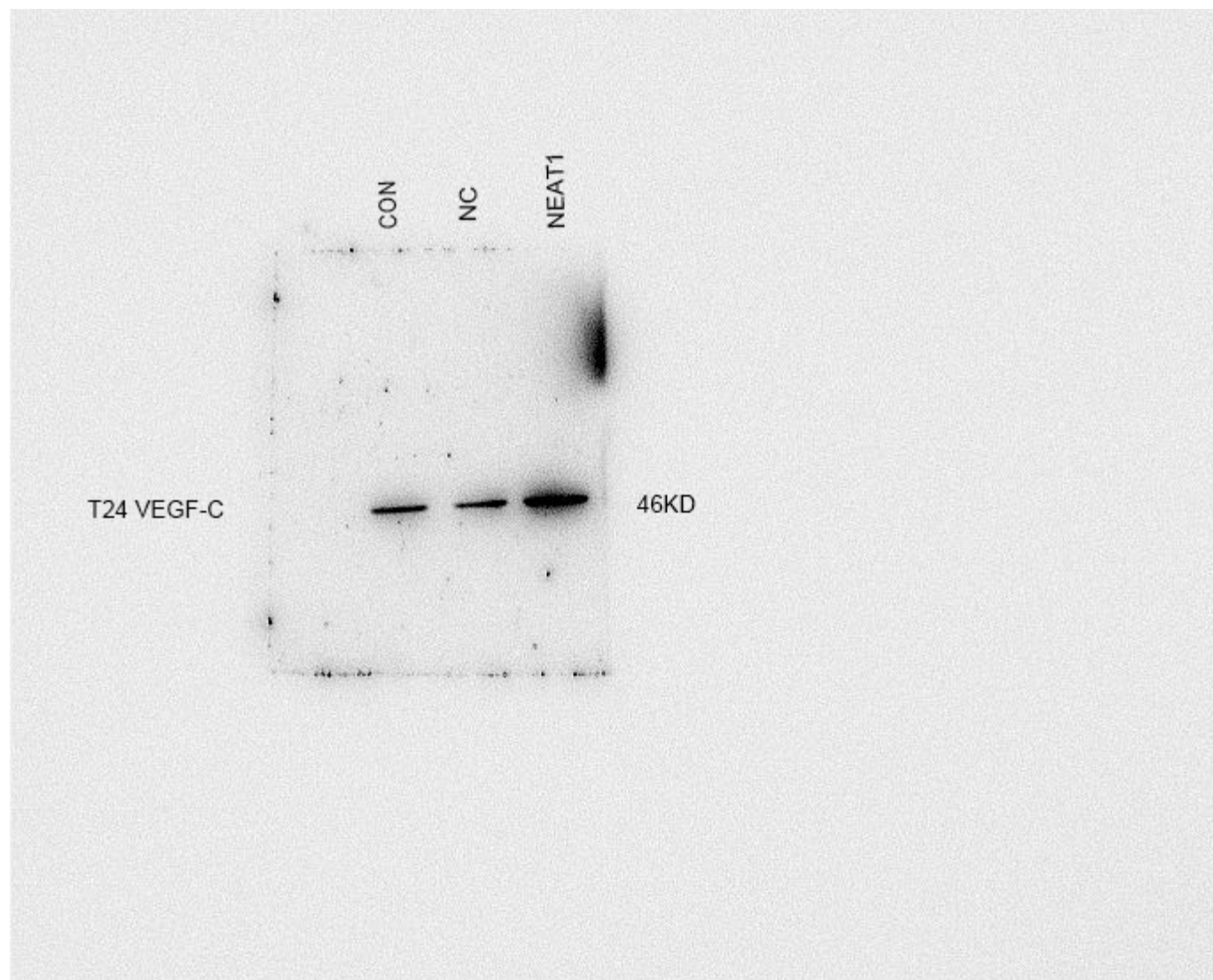

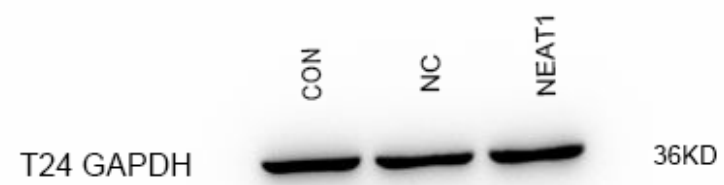

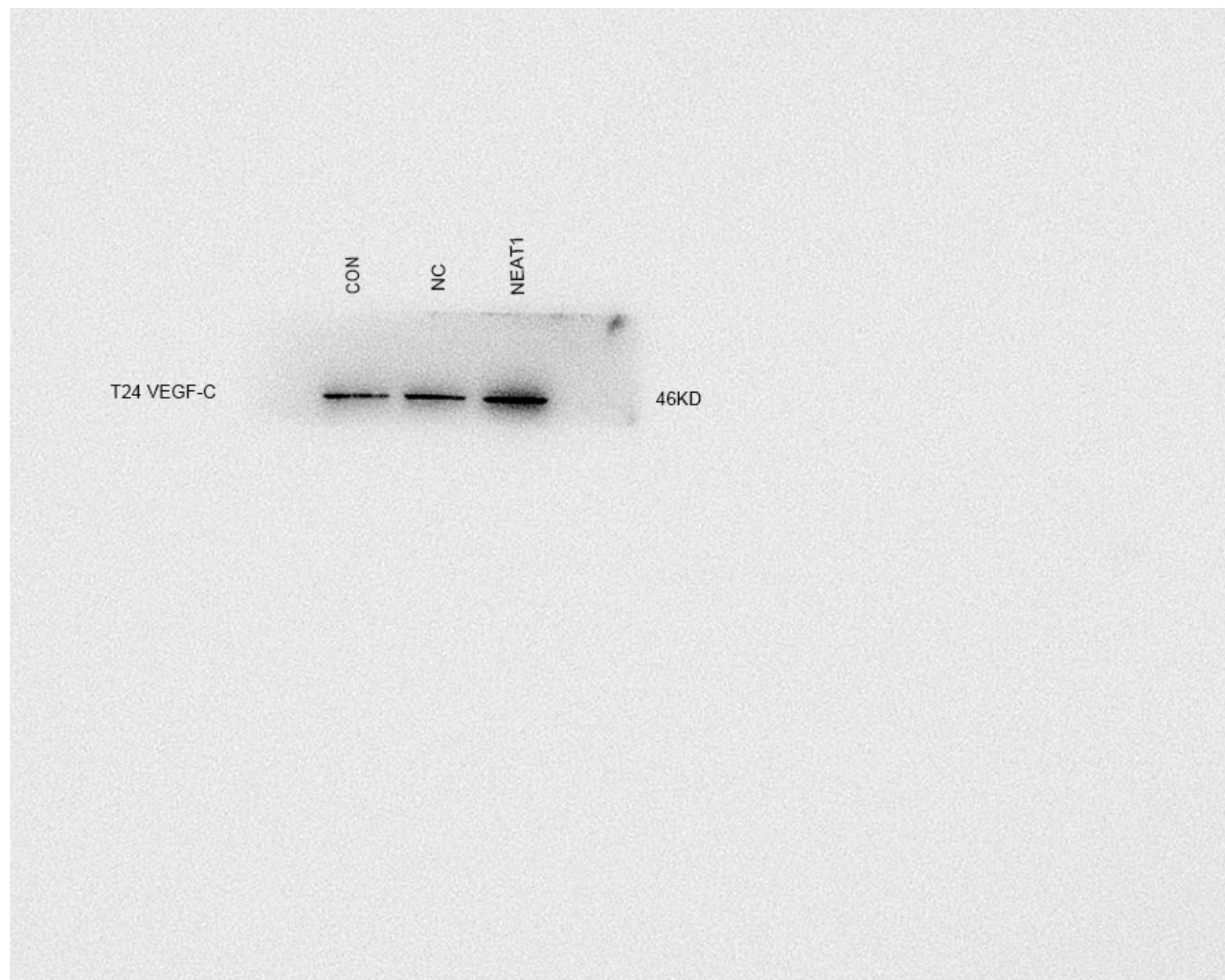

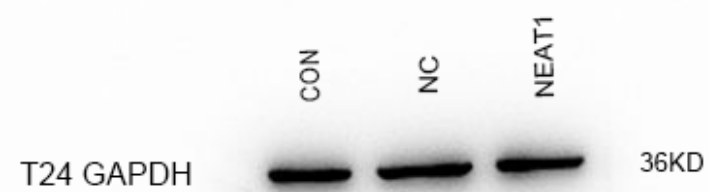

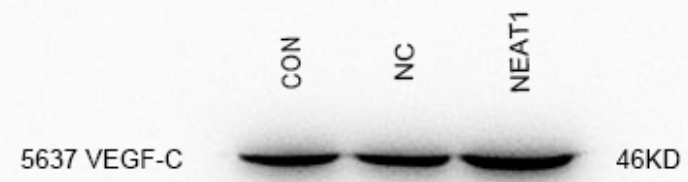

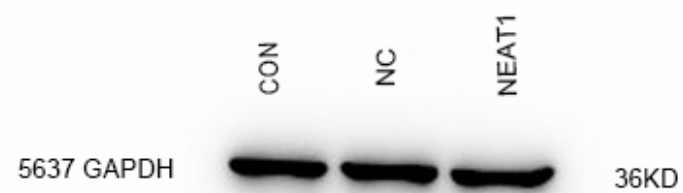

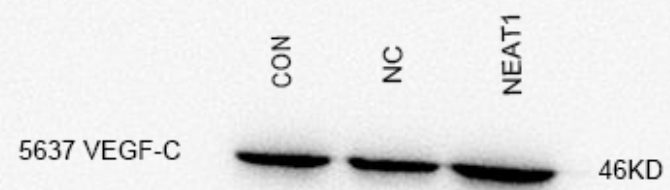

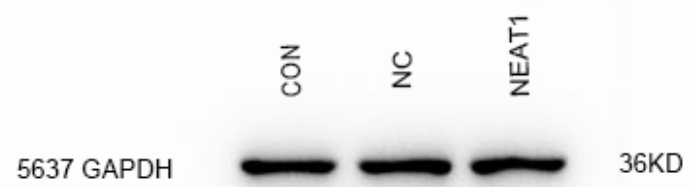

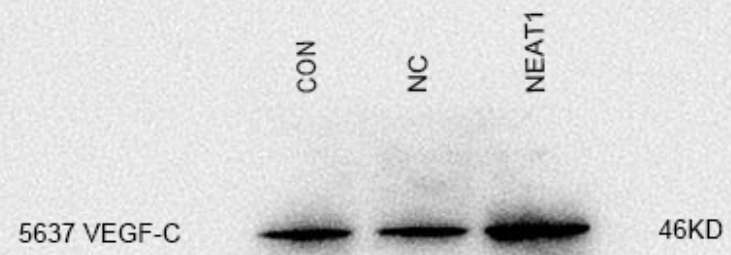

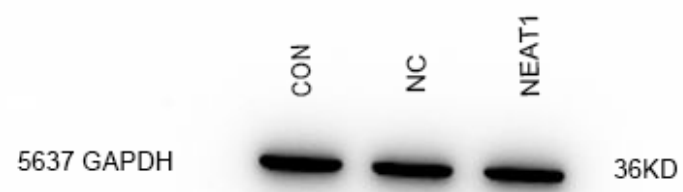

Supplement: Supplementary file 4 — Additional file 4. Original images of western blots for Fig. 5C. [file 12894_2022_1151_MOESM4_ESM.pdf]
