# Supplementary material for: NEAT1 promotes the malignant development of bladder cancer by regulating the miR-101/VEGF-C pathway in vitro and in vivo
Source: BMC Urol. 2022 Nov 25;22:193. doi: 10.1186/s12894-022-01151-z (PMC9700885; doi:10.1186/s12894-022-01151-z)

Supplementary Fig S3

Original western blots for Figure 3E

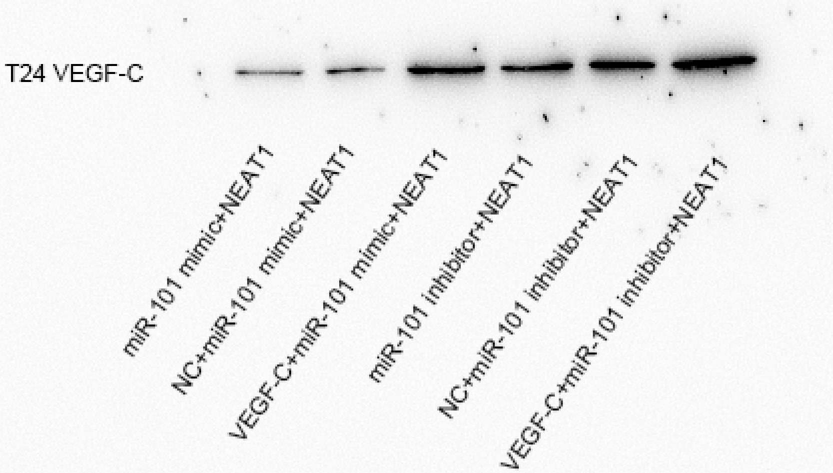

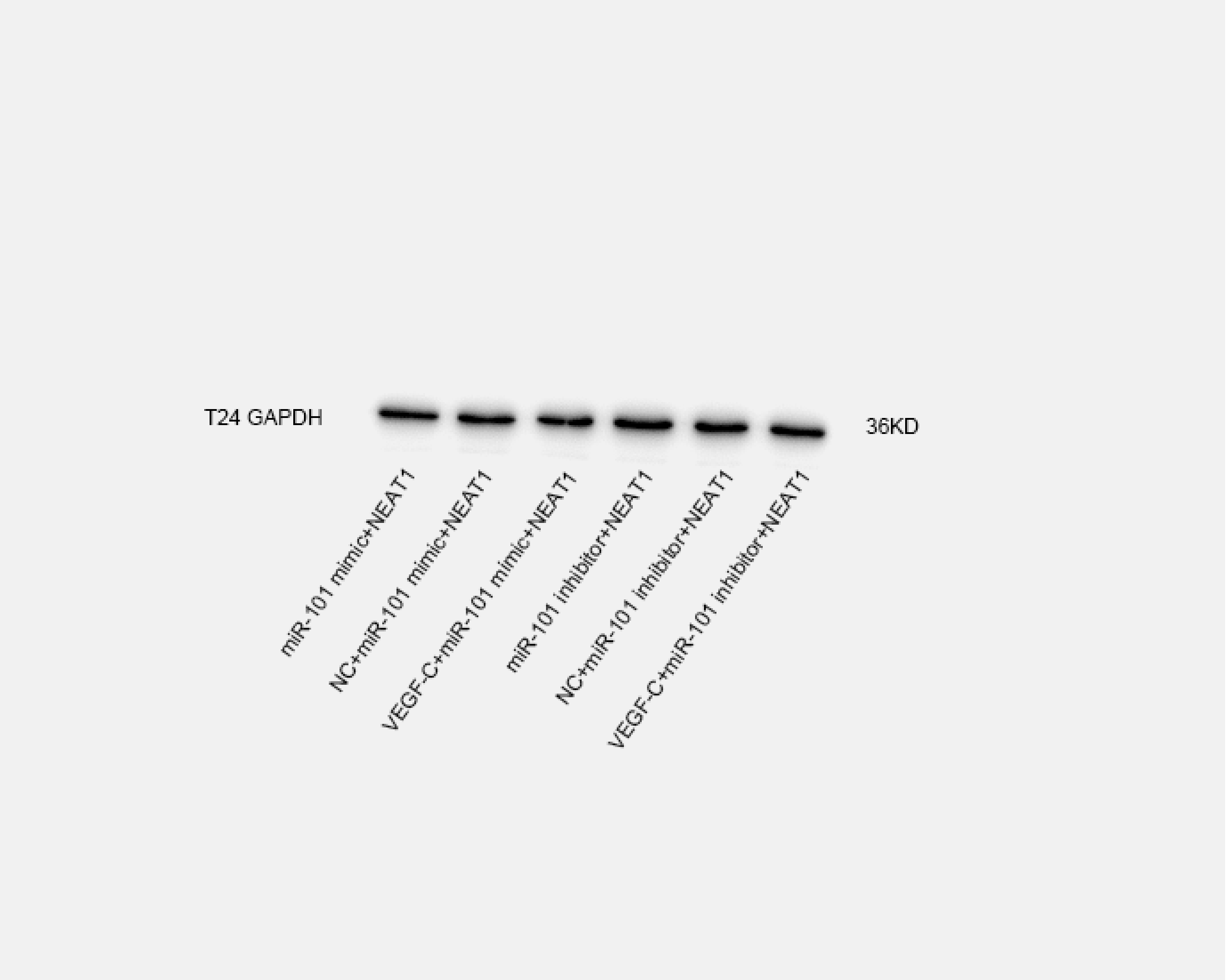
T24 GAPDH
36KD
miR-101 mimic+NEAT1
NC+miR-101 mimic+NEAT1
VEGF-C+miR-101 mimic+NEAT1
miR-101 inhibitor+NEAT1
NC+miR-101 inhibitor+NEAT1
VEGF-C+miR-101 inhibitor+NEAT1

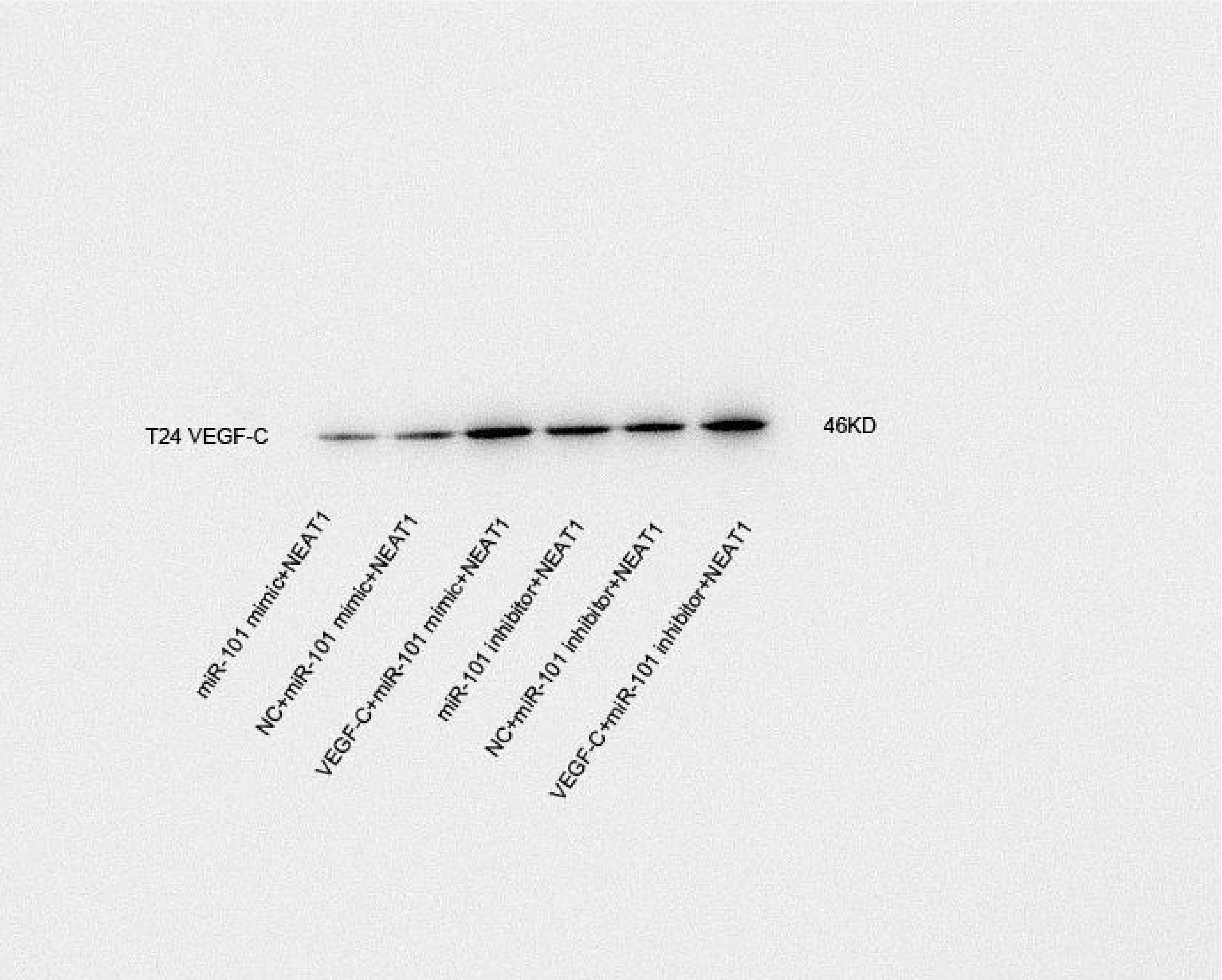
T24 VEGF-C
46KD
miR-101 mimic+NEAT1
NC+miR-101 mimic+NEAT1
VEGF-C+miR-101 mimic+NEAT1
miR-101 inhibitor+NEAT1
NC+miR-101 inhibitor+NEAT1
VEGF-C+miR-101 inhibitor+NEAT1

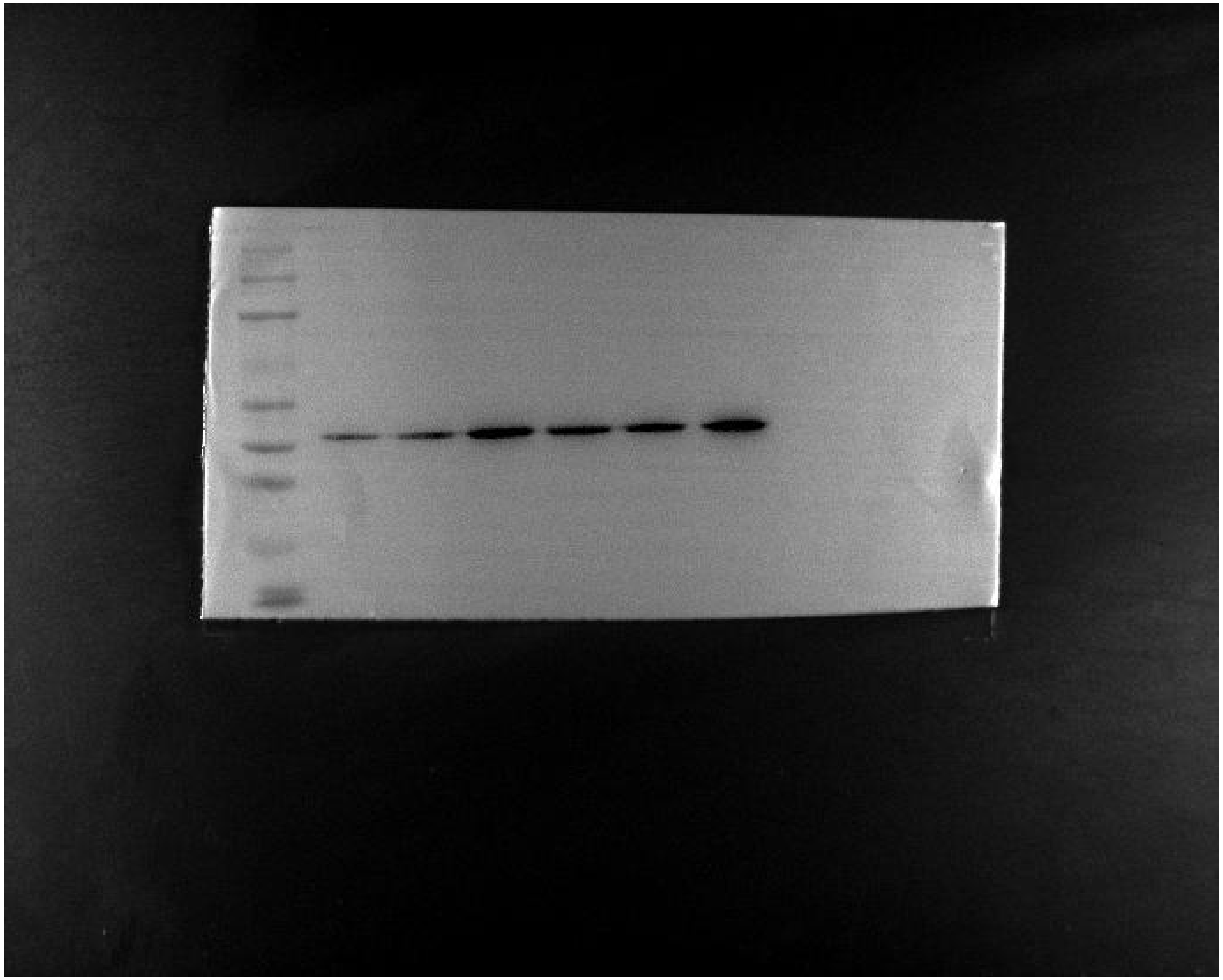

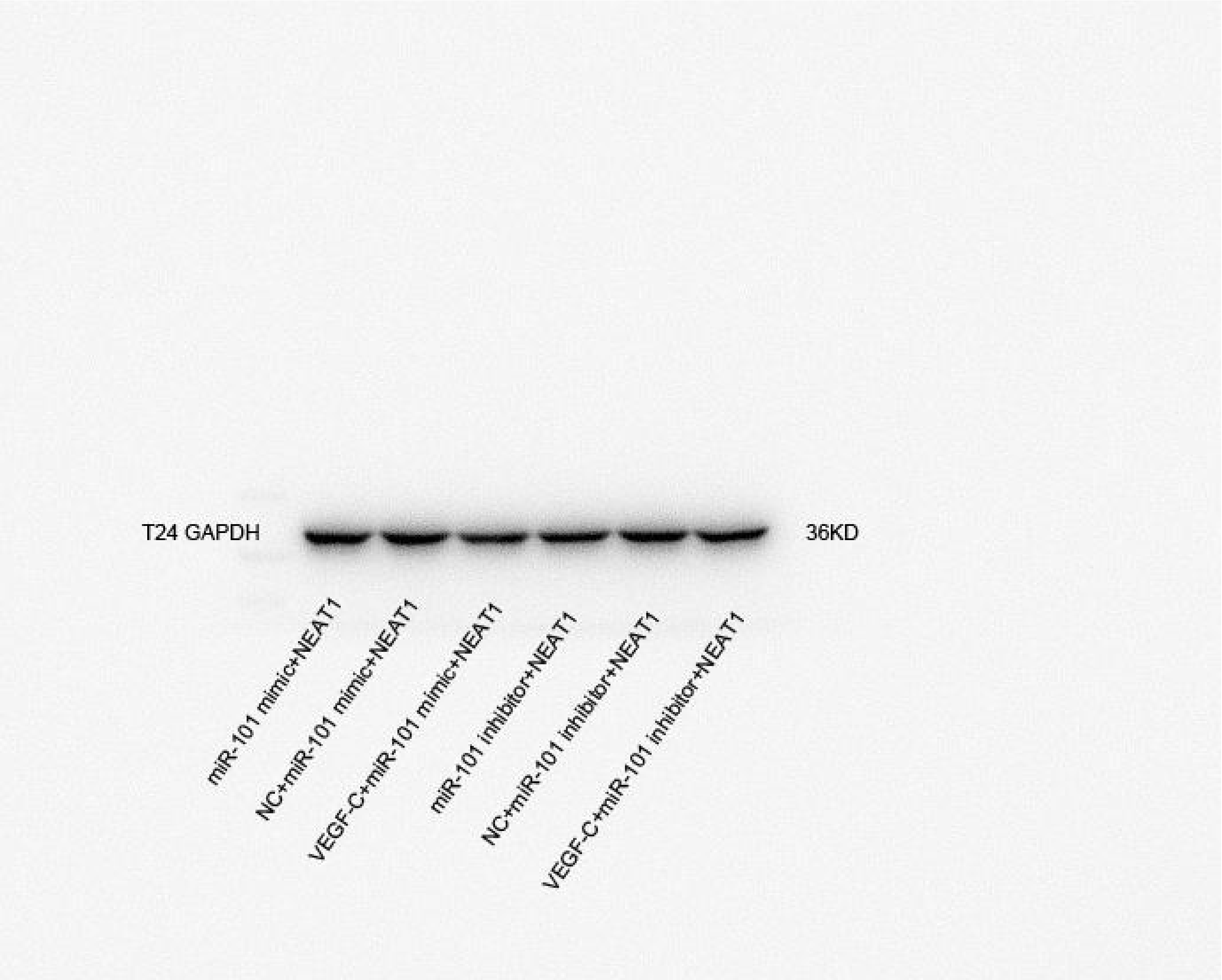
T24 GAPDH
36KD
miR-101 mimic+NEAT1
NC+miR-101 mimic+NEAT1
VEGF-C+miR-101 mimic+NEAT1
miR-101 inhibitor+NEAT1
NC+miR-101 inhibitor+NEAT1
VEGF-C+miR-101 inhibitor+NEAT1

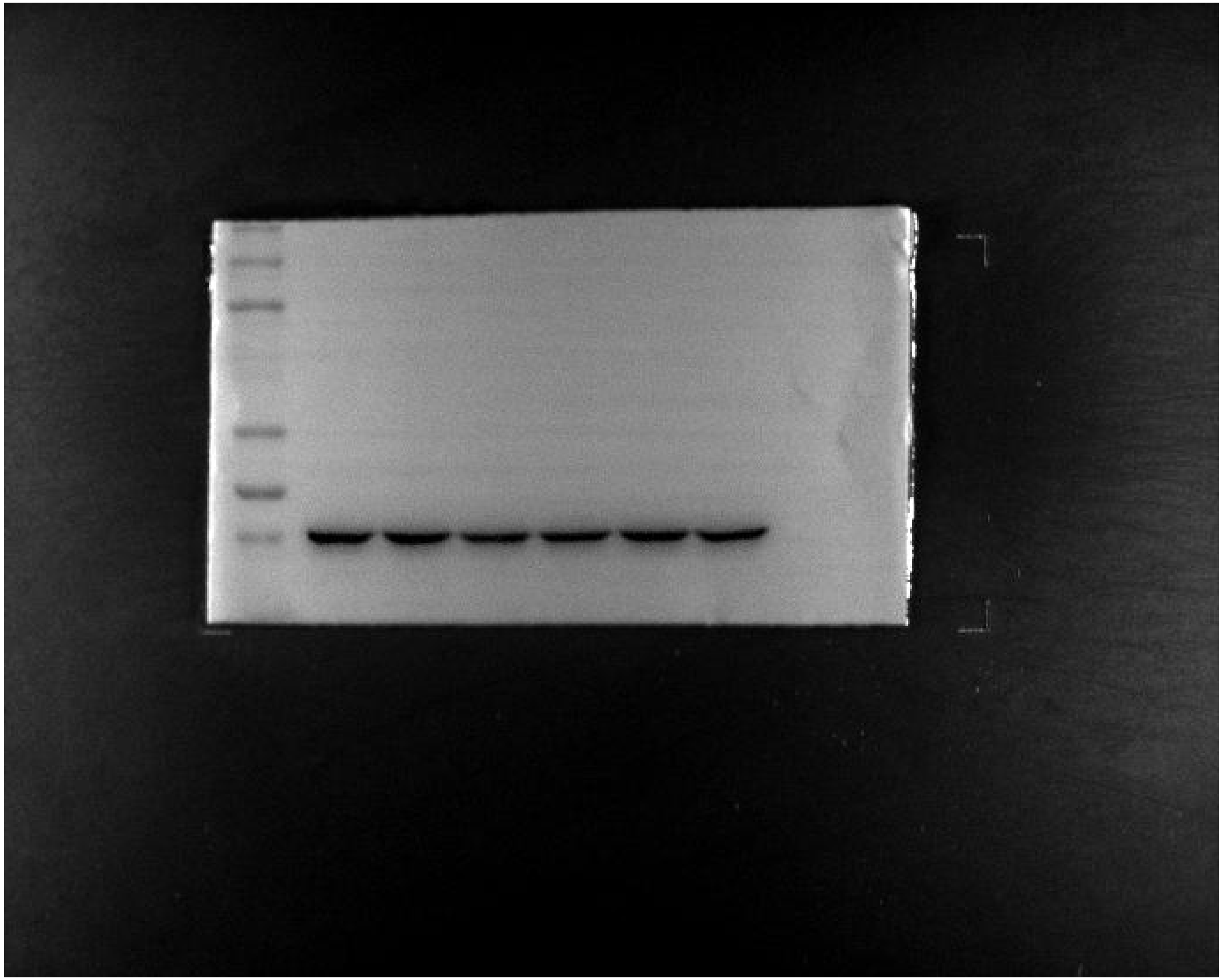

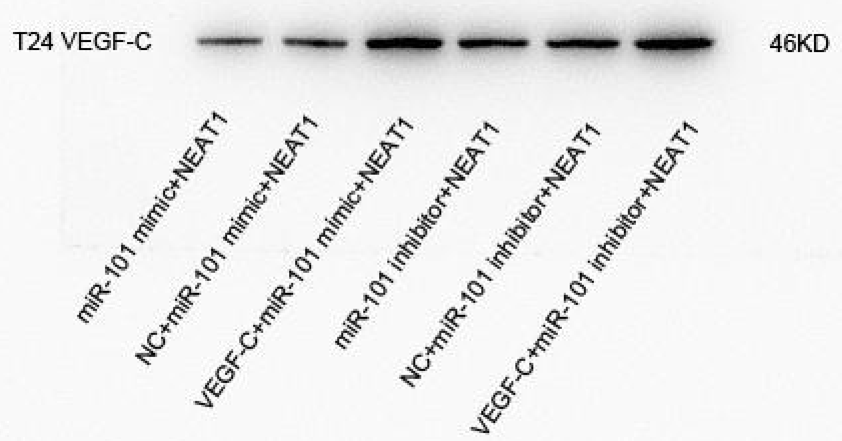
T24 VEGF-C
46KD
miR-101 mimic+NEAT1
NC+miR-101 mimic+NEAT1
VEGF-C+miR-101 mimic+NEAT1
miR-101 inhibitor+NEAT1
NC+miR-101 inhibitor+NEAT1
VEGF-C+miR-101 inhibitor+NEAT1

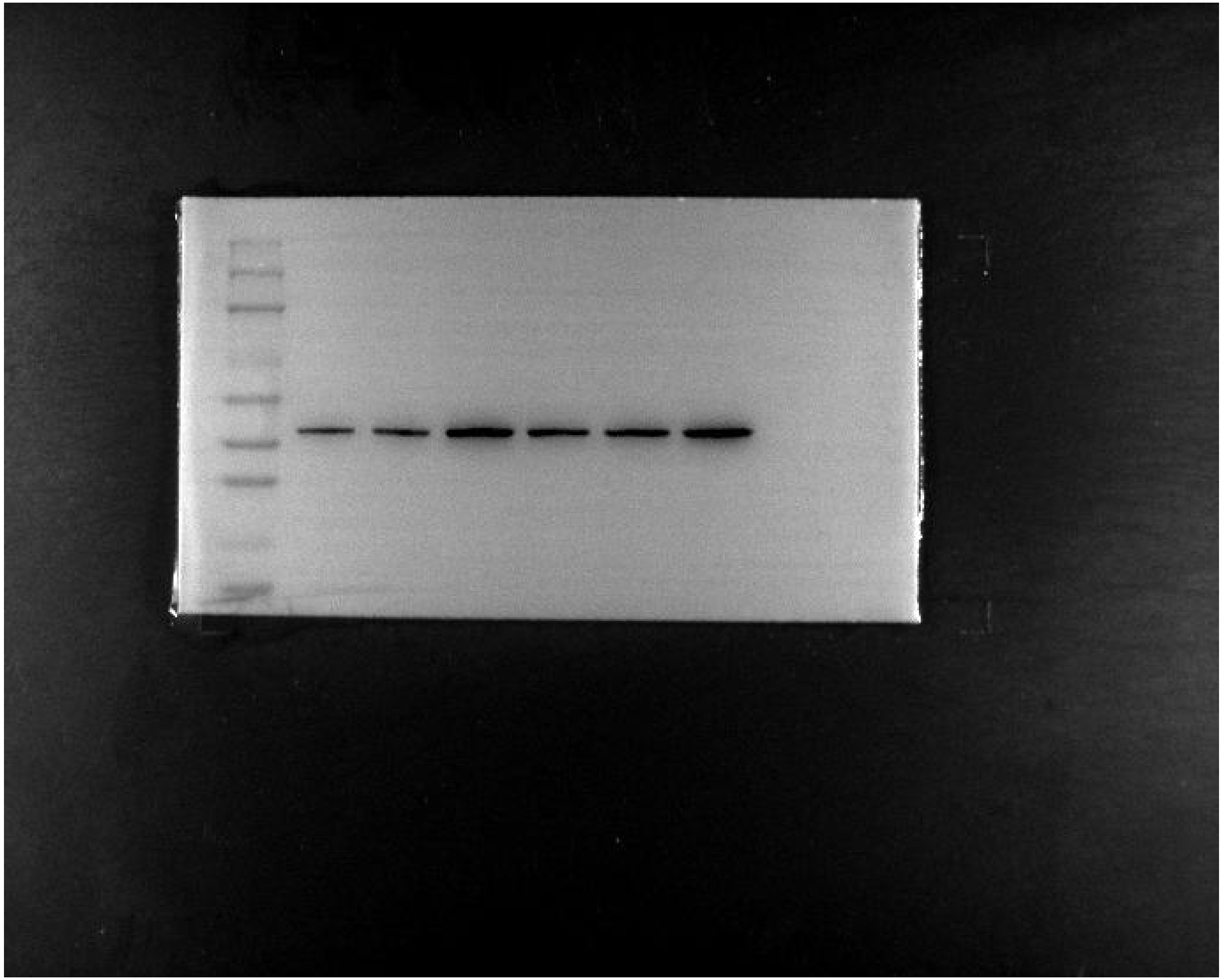

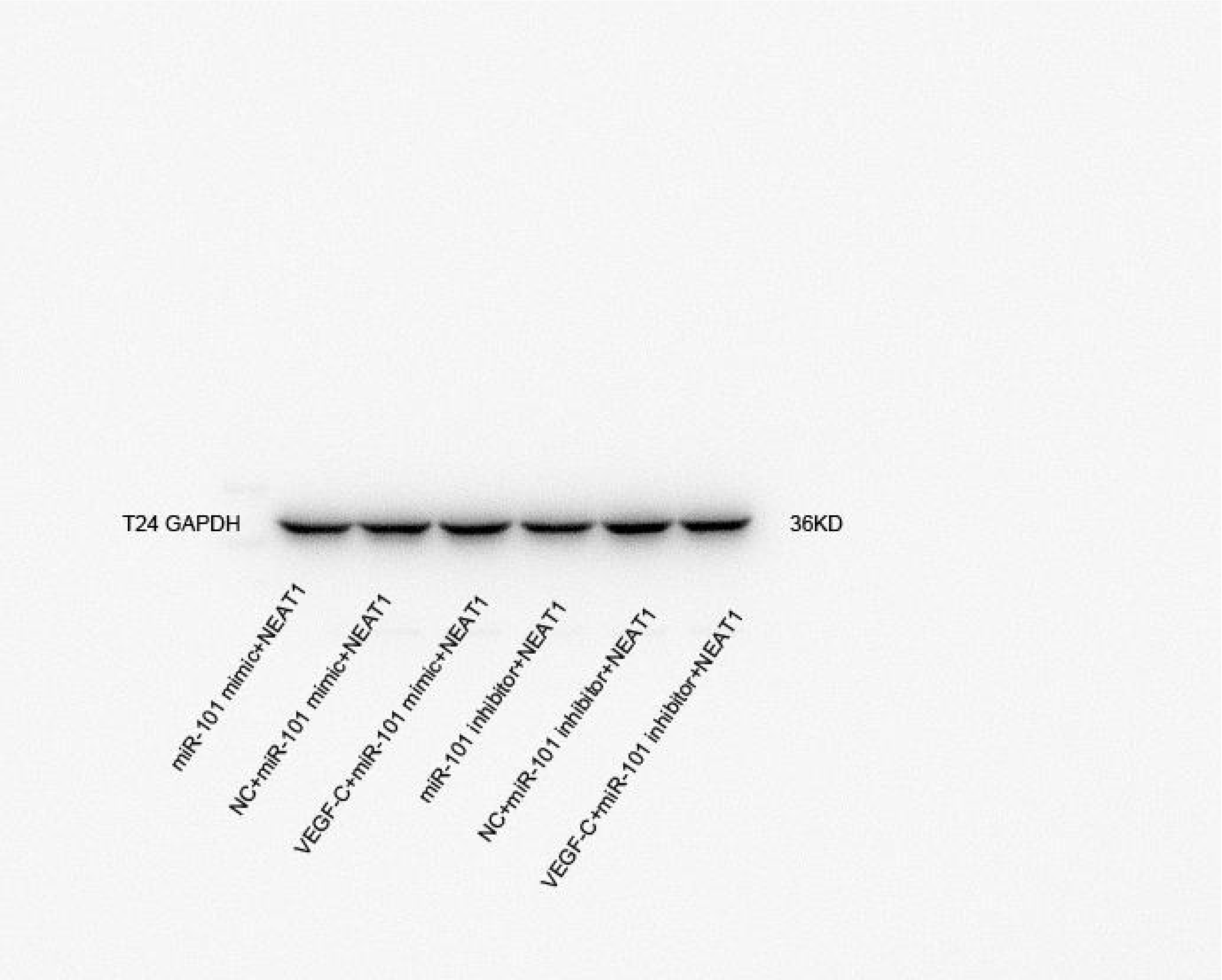
T24 GAPDH
36KD
miR-101 mimic+NEAT1
NC+miR-101 mimic+NEAT1
VEGF-C+miR-101 mimic+NEAT1
miR-101 inhibitor+NEAT1
NC+miR-101 inhibitor+NEAT1
VEGF-C+miR-101 inhibitor+NEAT1

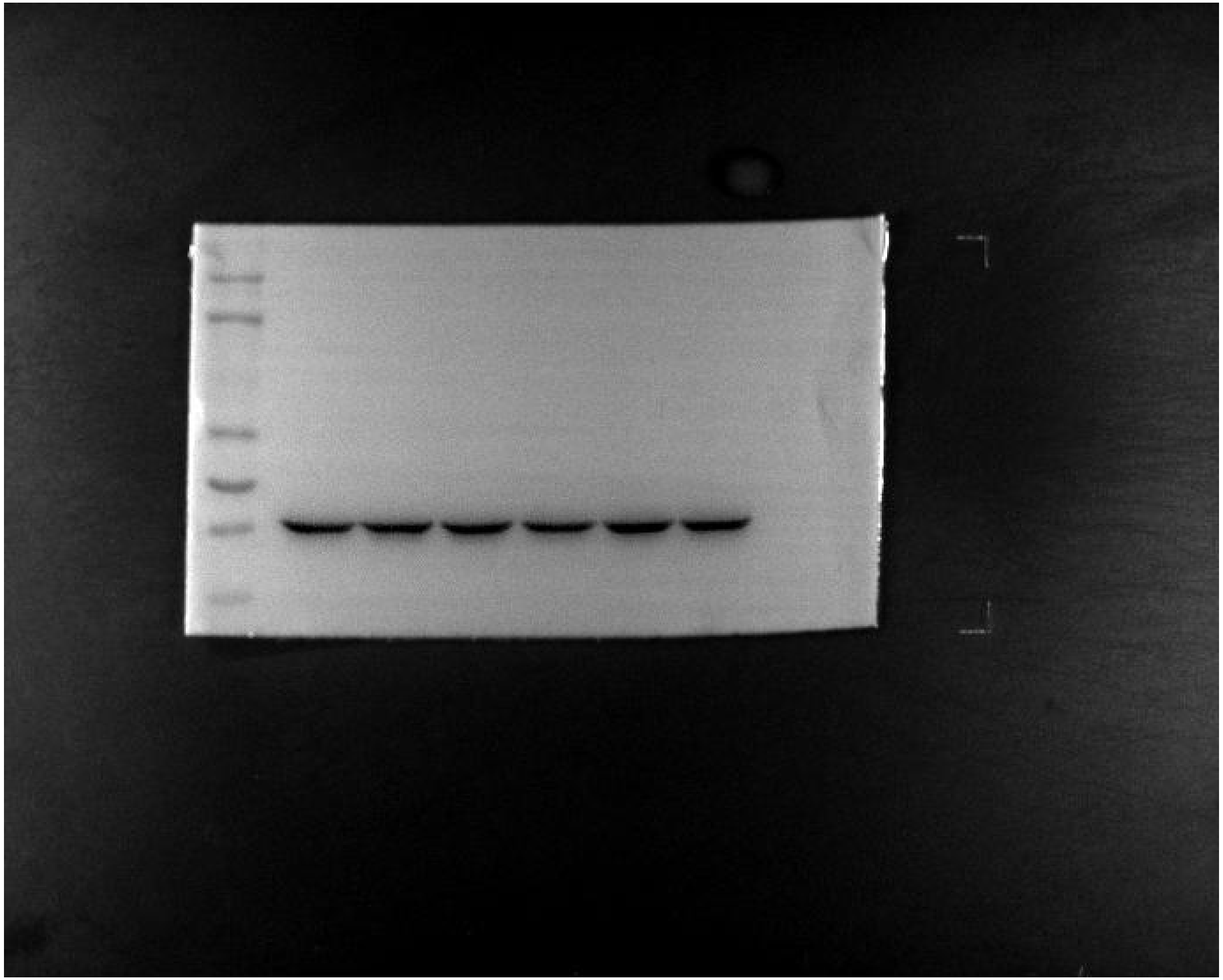

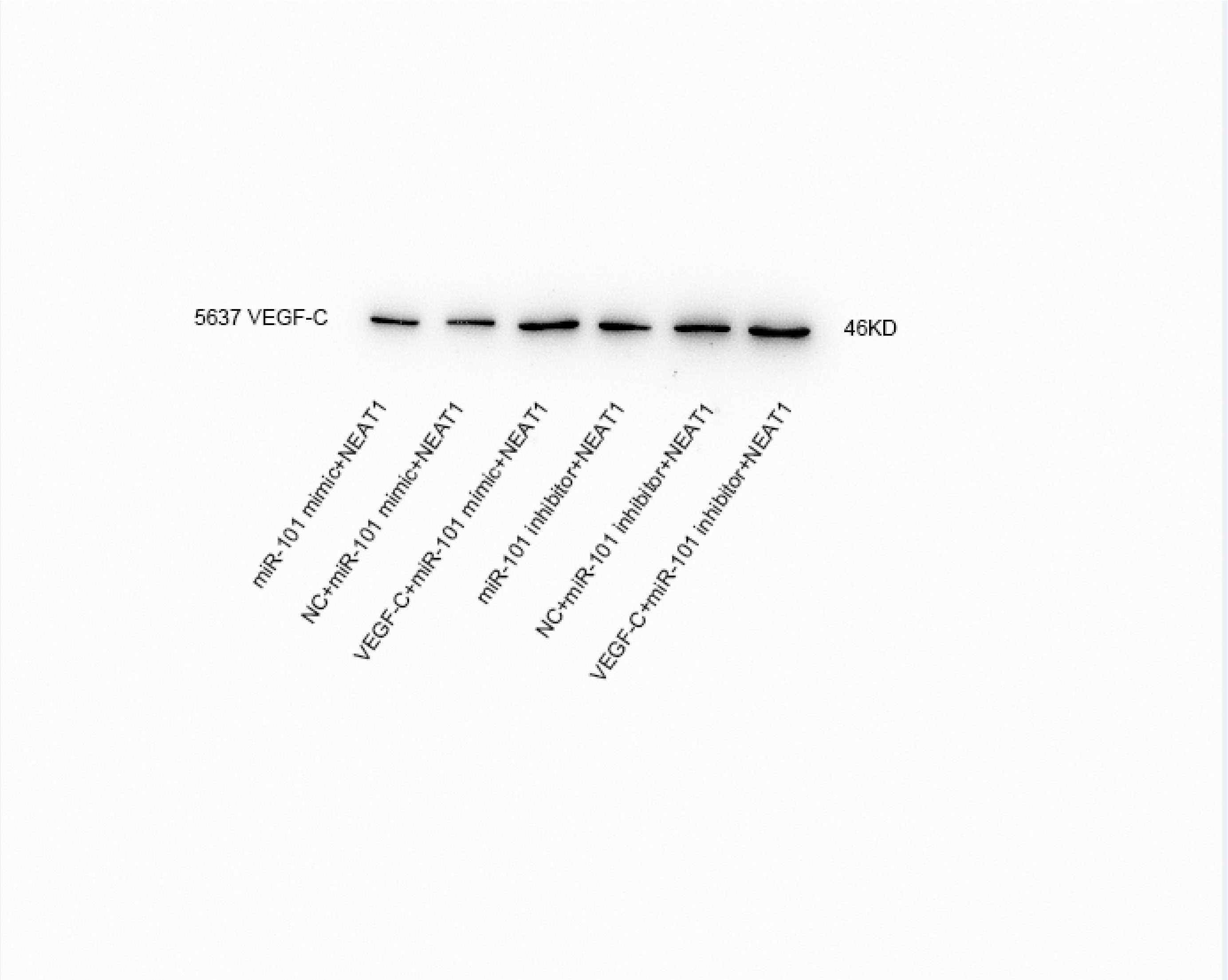
5637 VEGF-C
46KD
miR-101 mimic+NEAT1
NC+miR-101 mimic+NEAT1
VEGF-C+miR-101 mimic+NEAT1
miR-101 inhibitor+NEAT1
NC+miR-101 inhibitor+NEAT1
VEGF-C+miR-101 inhibitor+NEAT1

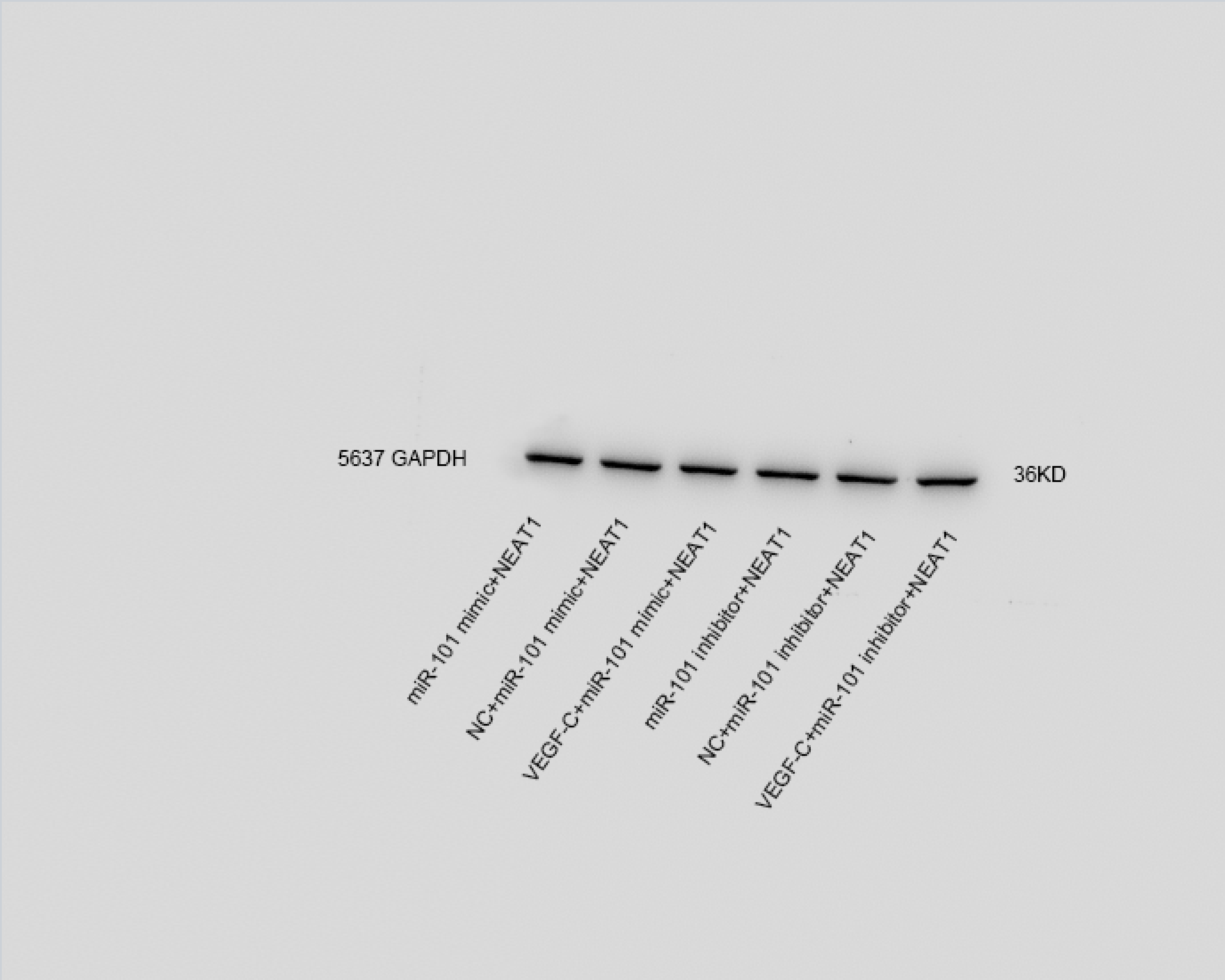
5637 GAPDH
36KD
miR-101 mimic+NEAT1
NC+miR-101 mimic+NEAT1
VEGF-C+miR-101 mimic+NEAT1
miR-101 inhibitor+NEAT1
NC+miR-101 inhibitor+NEAT1
VEGF-C+miR-101 inhibitor+NEAT1

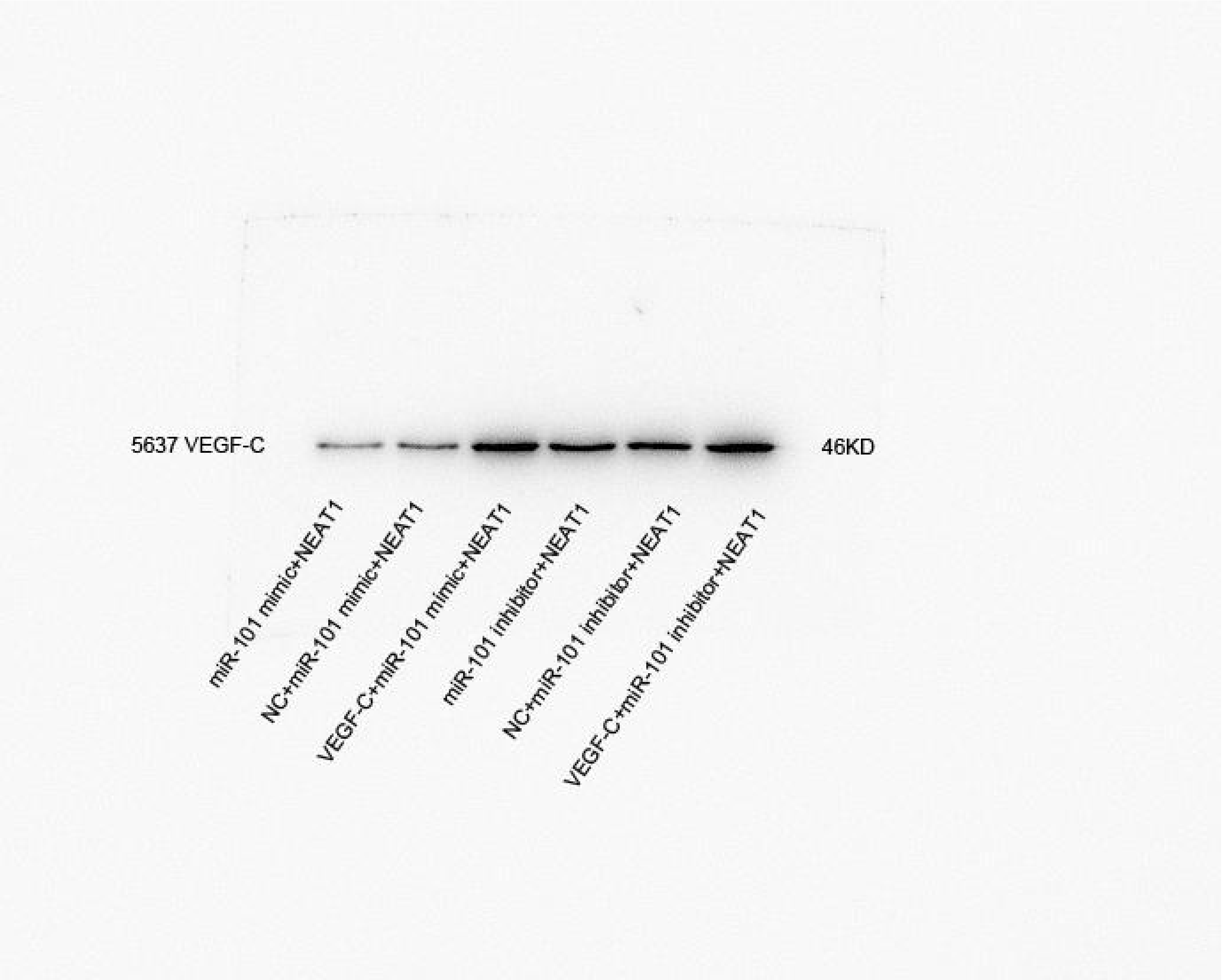
5637 VEGF-C
46KD
miR-101 mimic+NEAT1
NC+miR-101 mimic+NEAT1
VEGF-C+miR-101 mimic+NEAT1
miR-101 inhibitor+NEAT1
NC+miR-101 inhibitor+NEAT1
VEGF-C+miR-101 inhibitor+NEAT1

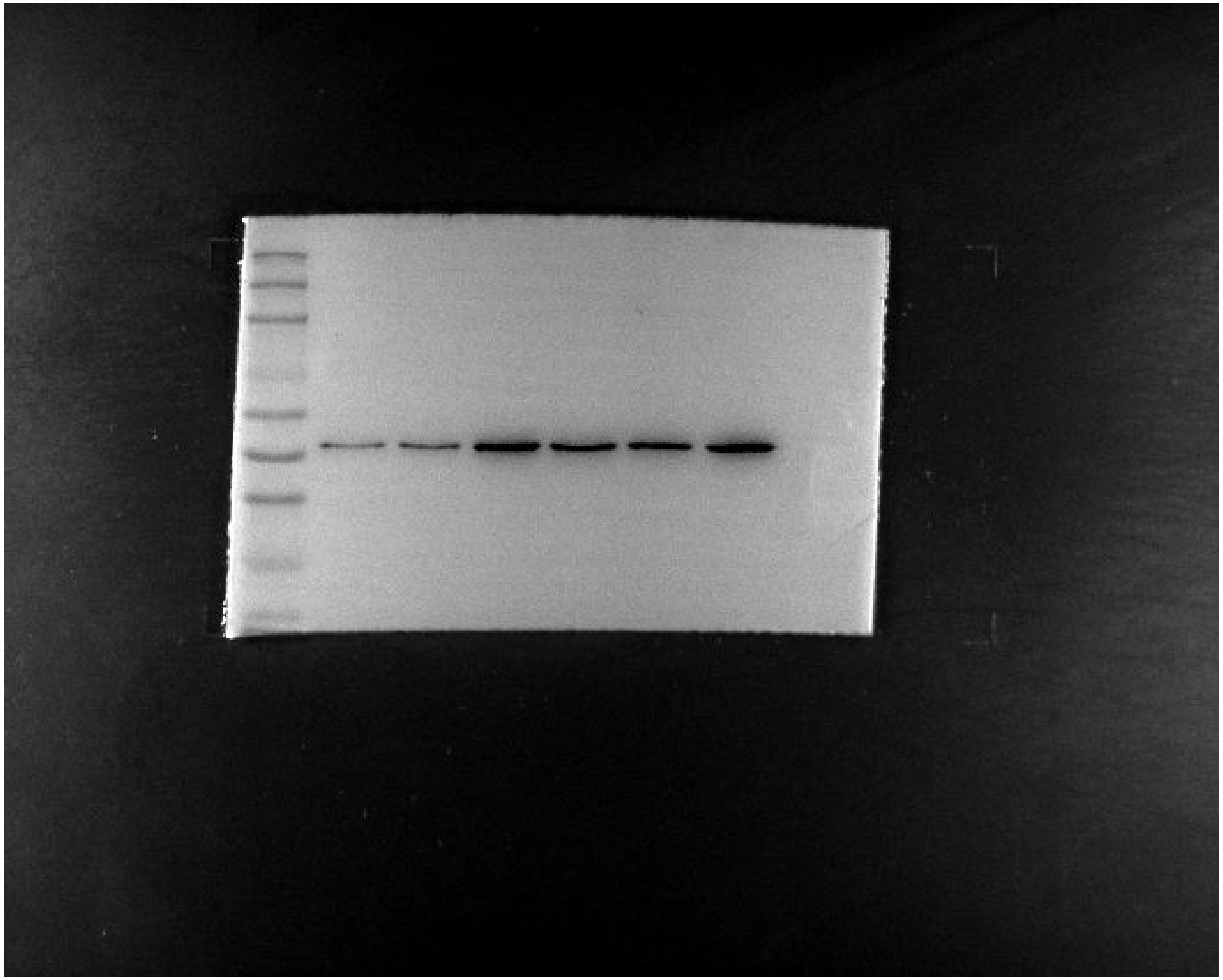

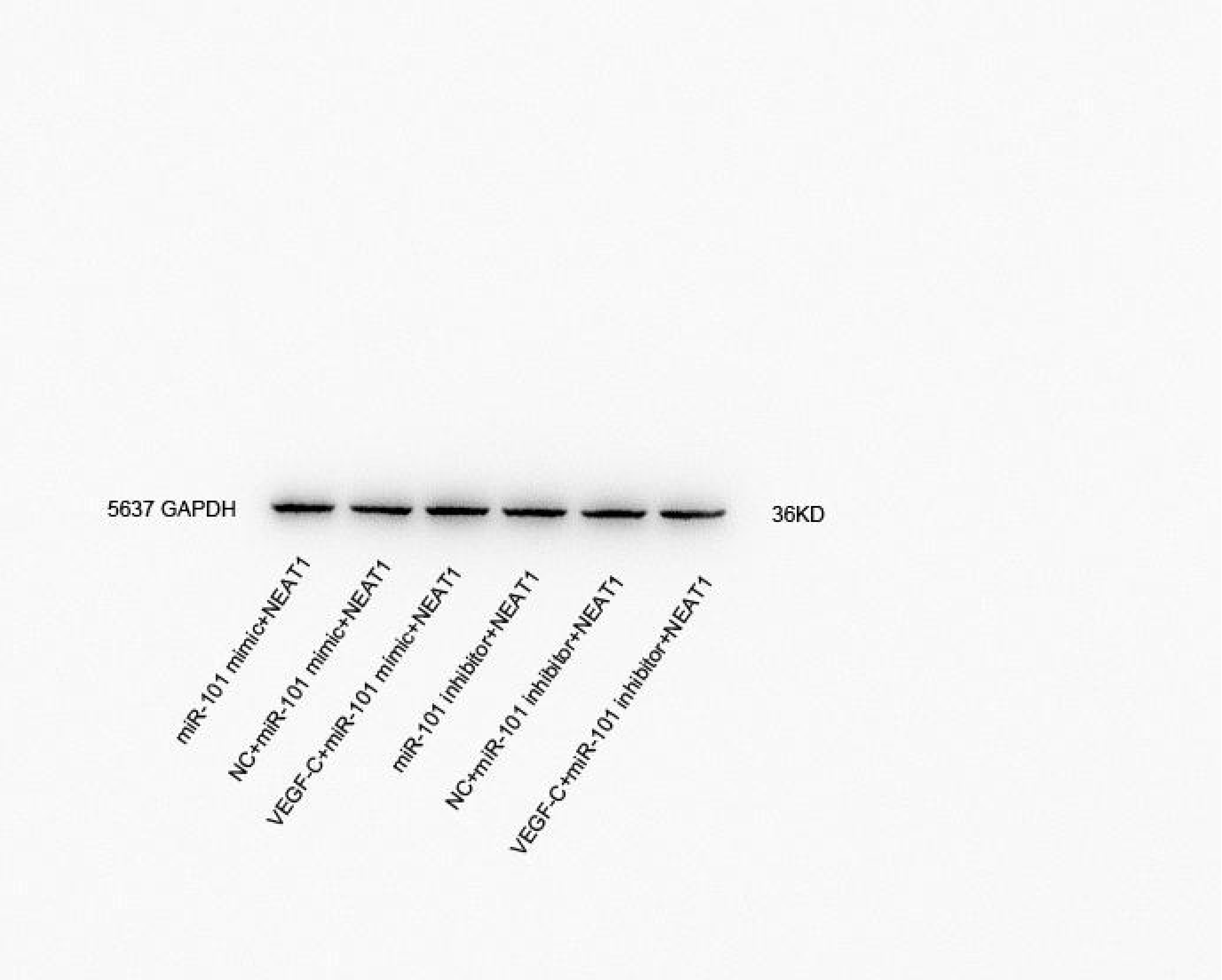
5637 GAPDH
36KD
miR-101 mimic+NEAT1
NC+miR-101 mimic+NEAT1
VEGF-C+miR-101 mimic+NEAT1
miR-101 inhibitor+NEAT1
NC+miR-101 inhibitor+NEAT1
VEGF-C+miR-101 inhibitor+NEAT1

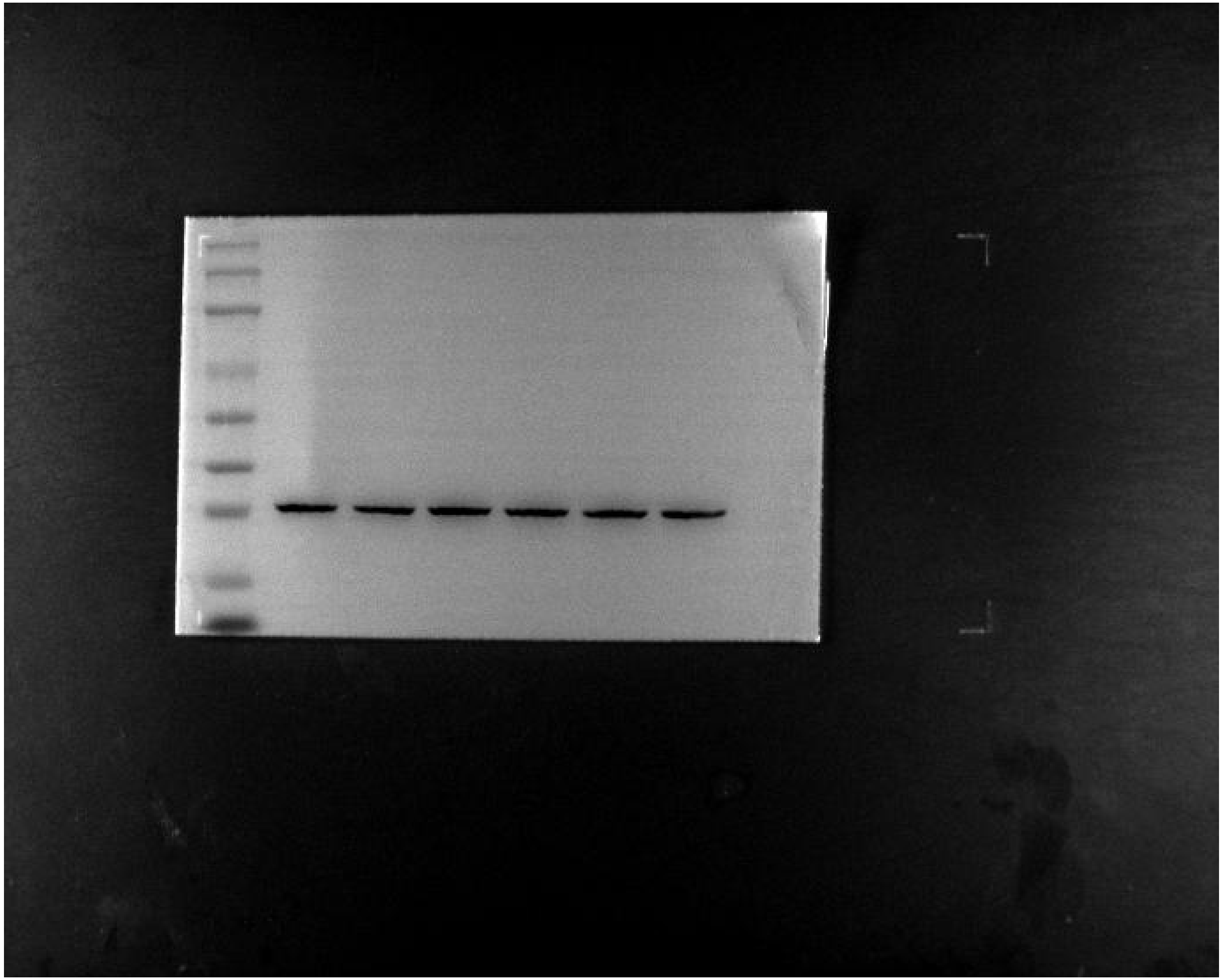

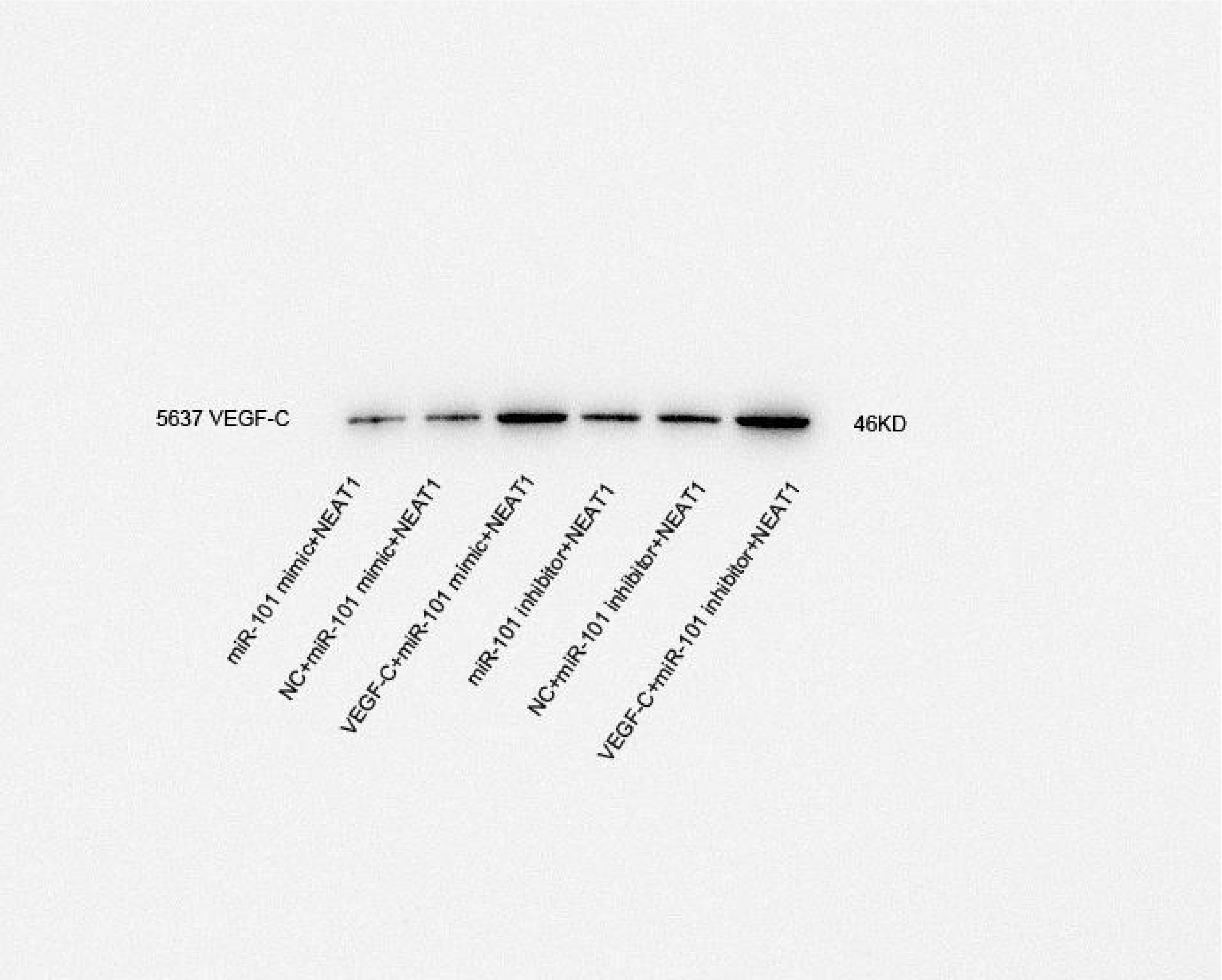
5637 VEGF-C
46KD
miR-101 mimic+NEAT1
NC+miR-101 mimic+NEAT1
VEGF-C+miR-101 mimic+NEAT1
miR-101 inhibitor+NEAT1
NC+miR-101 inhibitor+NEAT1
VEGF-C+miR-101 inhibitor+NEAT1

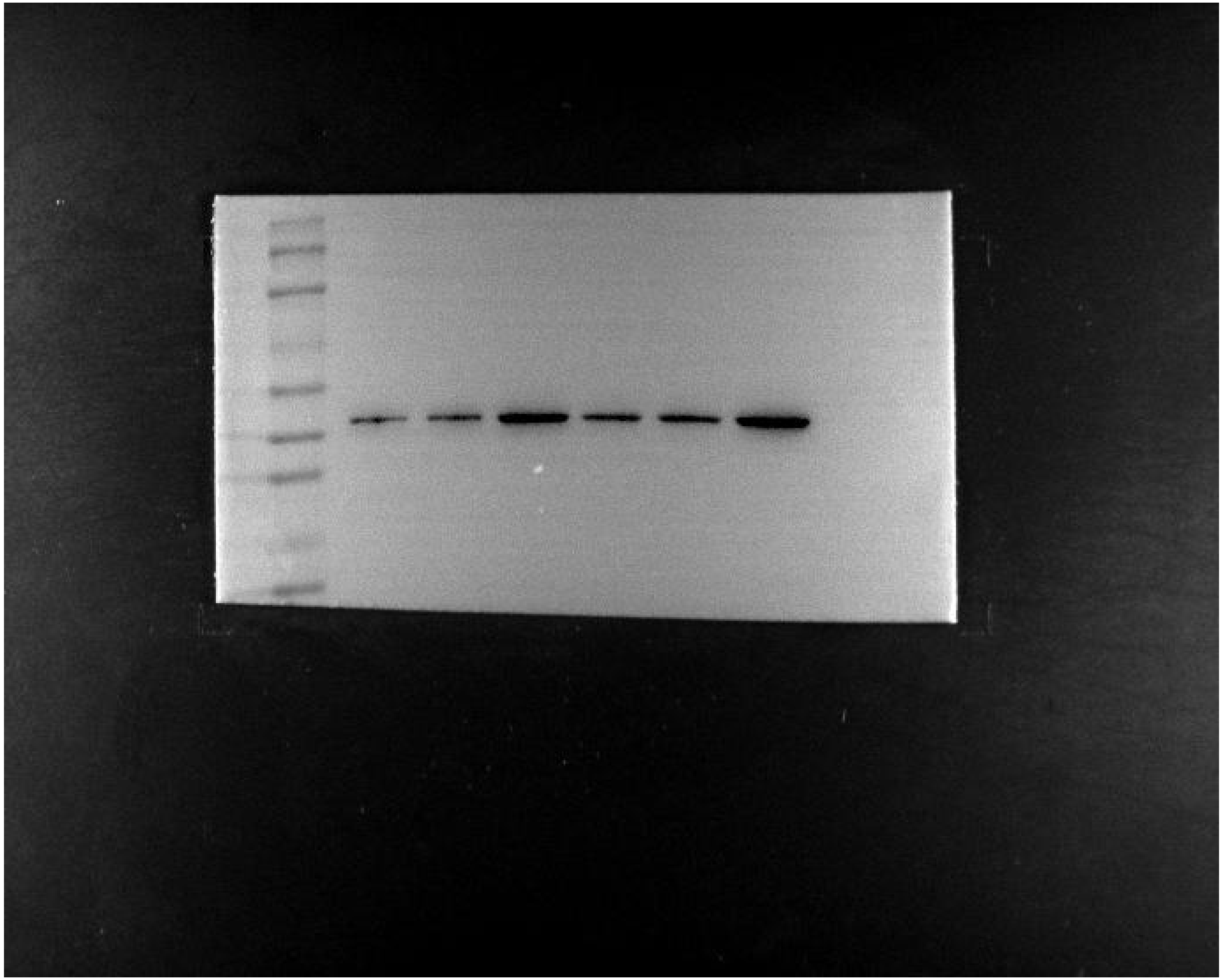

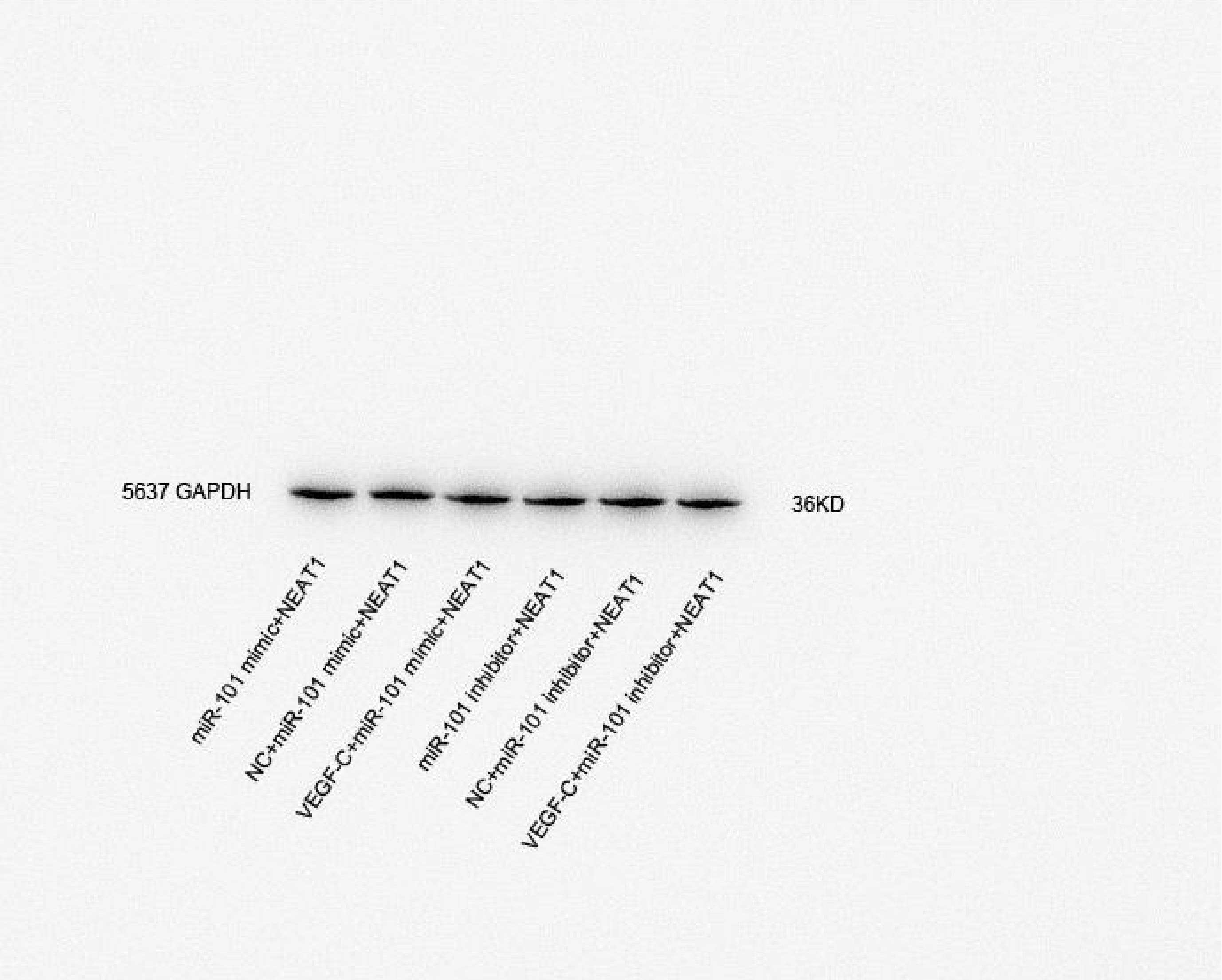
5637 GAPDH
36KD
miR-101 mimic+NEAT1
NC+miR-101 mimic+NEAT1
VEGF-C+miR-101 mimic+NEAT1
miR-101 inhibitor+NEAT1
NC+miR-101 inhibitor+NEAT1
VEGF-C+miR-101 inhibitor+NEAT1

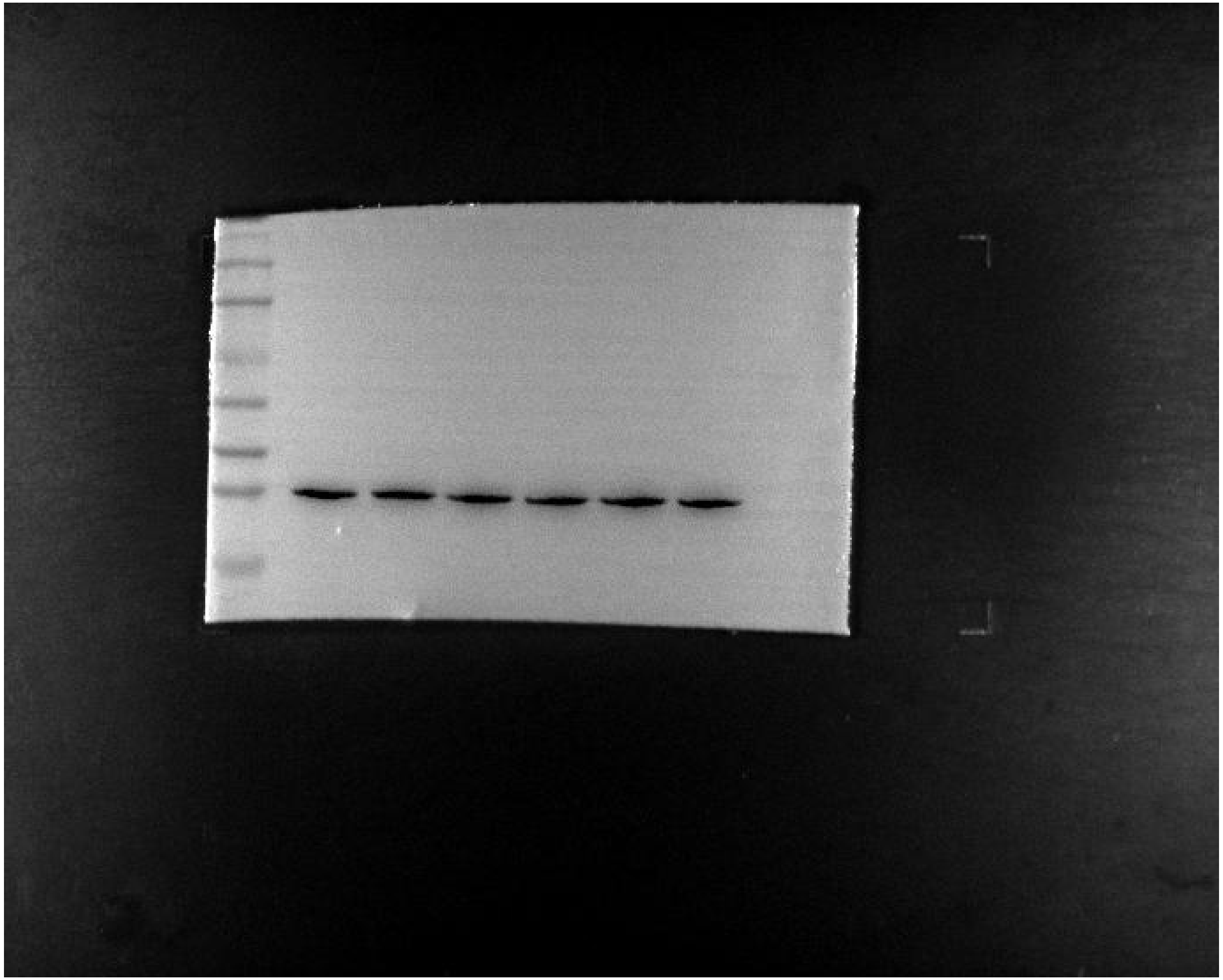

Supplement: Supplementary file 3 — Additional file 3. Original images of western blots for Fig. 3E. [file 12894_2022_1151_MOESM3_ESM.pdf]
